# Supplementary material for: Prunus Knotted-like Genes: Genome-Wide Analysis, Transcriptional Response to Cytokinin in Micropropagation, and Rootstock Transformation
Source: Int J Mol Sci. 2023 Feb 3;24(3):3046. doi: 10.3390/ijms24033046 (PMC9918302; doi:10.3390/ijms24033046)
Supplement: Supplementary file 1 [file ijms-24-03046-s001.zip › Supplementary Figures/Figure S1. Cytokinin binding sites on CK-responsive genes.pdf]

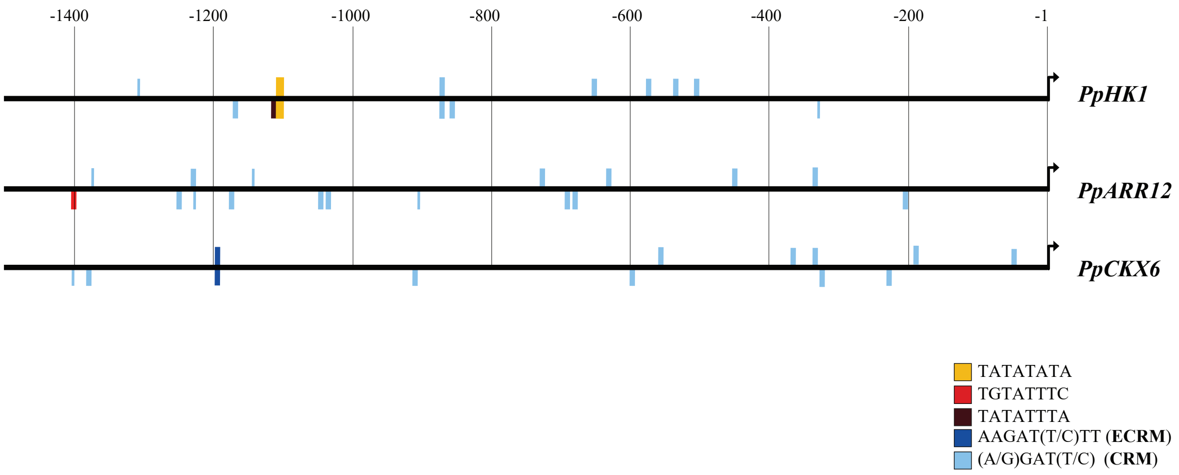

Figure S1. Cytokinin binding sites on CK-responsive genes.
The scheme draw the position of CK binding sites in the 1500 bp region upstream the start codon (arrows) of genes used as CK-responsive markers. The binding sites included the experimentally-determined extended (ECRM; blue) and core (CRM; light blue) motifs [38], and octameric sequences (yellow/orange/brown) which were found enriched in promoters of CK-responsive genes [39].
