# Supplementary material for: Prunus Knotted-like Genes: Genome-Wide Analysis, Transcriptional Response to Cytokinin in Micropropagation, and Rootstock Transformation
Source: Int J Mol Sci. 2023 Feb 3;24(3):3046. doi: 10.3390/ijms24033046 (PMC9918302; doi:10.3390/ijms24033046)
Supplement: Supplementary file 1 [file ijms-24-03046-s001.zip › Supplementary Figures/Figure S2. Regeneration and transformation.pdf]

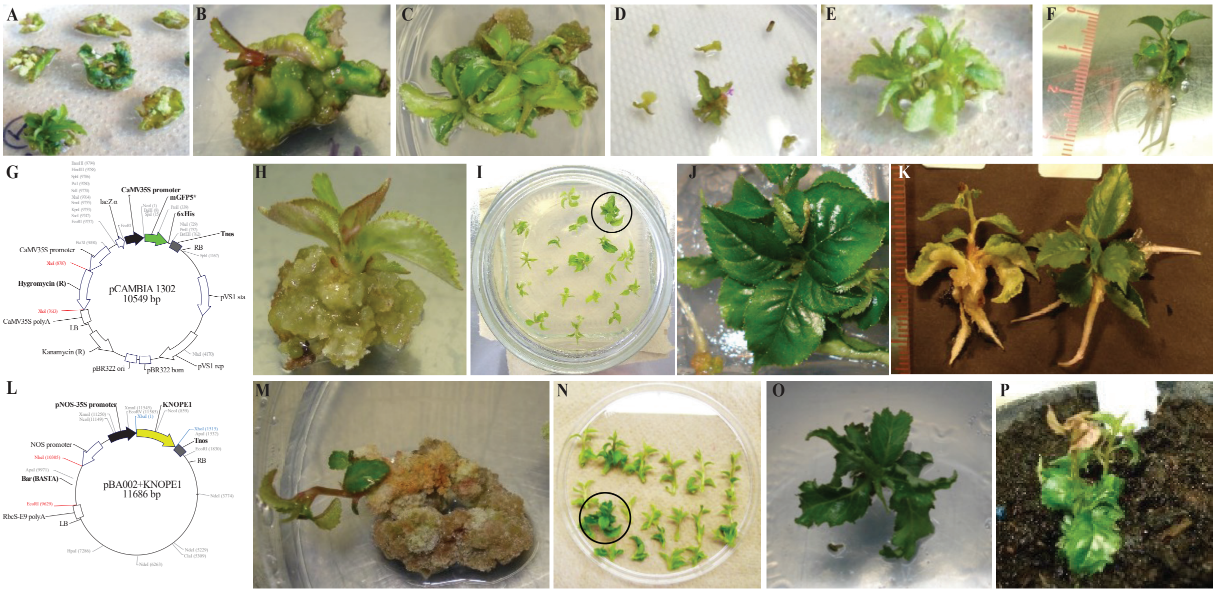

Figure S2. Regeneration and transformation of Gisela 6 explants.

Indirect regeneration from leaf (**A-C**) and petiole (**D-F**) explants. Calluses formed at the wound sites of explants (**A, D**) and single (**B, D**) and multiple (**C, E**) shoots developed. Petiole were not used in transformation experiments due to a lower number of shoots *per* explant.

GFP transfer process (**G-K**). The *pCAMBIA1302* vector (**G**) conferring resistance to HYG and synthetizing the GFP was used. Restriction sites used to excise the *HPTII* probe for Southern blot analysis are in red. Example of shoot regenerated from HYG resistant callus (**H**) and (**I**) 2nd round of selection. A putative resistant clone is circled. **J**) Putative *GFP*-transformed clone upon selective media. **K**) *GFP*-transformed rooted clones. Left, HYG sensitive; right, putative transformed HYG+ clone.

KNOPE1 transfer process (**L-P**). *KNOPE1* was cloned (restriction enzymes in blue) into the *pBA002* vector (**L**) conferring resistance to PPT. Restriction sites used to excise the *BAR* probe for Southern blot analysis are in red. **M**) Example of indirect organogenesis from PPT resistant callus and 2nd round of selection. A putative resistant clone is circled. **O**) *KNOPE1*-transformed clone showing altered leaf margins and stunt phenotype. **P**) *KNOPE1* clone adapted on soil.
