# Supplementary material for: Prunus Knotted-like Genes: Genome-Wide Analysis, Transcriptional Response to Cytokinin in Micropropagation, and Rootstock Transformation
Source: Int J Mol Sci. 2023 Feb 3;24(3):3046. doi: 10.3390/ijms24033046 (PMC9918302; doi:10.3390/ijms24033046)
Supplement: Supplementary file 1 [file ijms-24-03046-s001.zip › Supplementary Figures/Figure S3. Southern blots.pdf]

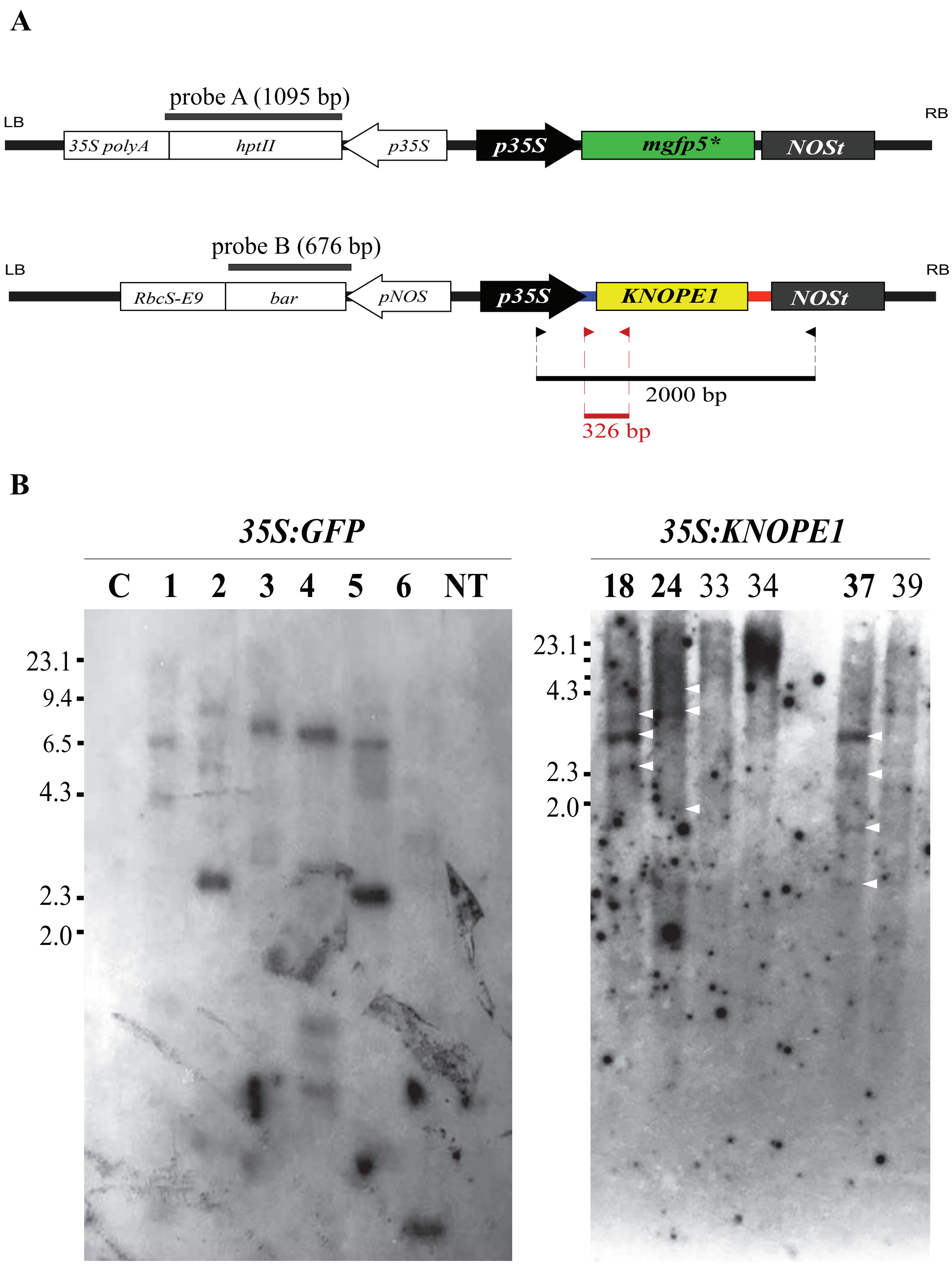

Figure S3

Southern blot analyses by EcoRI-digested gDNA and hybridised with A and B probes shown in panel A. C, control; NT, non-transformed DNA plant. White arrows point at signal bands that were counted to declare confidently the occurrence of multiple T-DNA copies. Black dots were caused by debris and impurities.
