# Supplementary material for: Prunus Knotted-like Genes: Genome-Wide Analysis, Transcriptional Response to Cytokinin in Micropropagation, and Rootstock Transformation
Source: Int J Mol Sci. 2023 Feb 3;24(3):3046. doi: 10.3390/ijms24033046 (PMC9918302; doi:10.3390/ijms24033046)
Supplement: Supplementary file 1 [file ijms-24-03046-s001.zip › Supplementary Tables/Table S2. Protein group features and analysis of variable amino acid stretches.pdf]

Table S2. Protein group features and analysis of variable amino acid stretches.

Group	Molecular weights (kDa)		Isoelectric point		Variable amino acid stretches						
	Range	cv[1]	Range	cv	Pos.	Domain	CS[2]	Variants[3]	Ref/Alt[4]	ST[5]	SIFT[6]
STMlike1	36.6-37.4	0.5%	6.5-7.3	3.1%	11	N-term.	0.73	SNNNNSNNSN	Ser/Asn	S	1.00
					71	N-term.	0.71	EKKKKEEEEE	Lys/Glu	D	1.00
					196	N-term.	0.73	MMKKKMMMMM	Met/Lys	D	1.00
STMlike2	40.3-42.9	1.9%	6.8-7.0	0.9%	161	KNOX1	0.71	AAAAASSSA	Ala/Ser	D	0.87
KNOPE1	44.0-44.4	0.4%	6.6-6.6	0.0%	38	N-term.	0.74	MMMMMIMMII	Met/Ile	S	0.95
					59	N-term.	0.74	AAAAAGAAGG	Ala/Gly	S	0.63
					244	C-term.	0.71	LLLLLIILII	Leu/Ile	S	1.00
KNOPE2	40.0-40.8	0.7%	4.8-5.1	1.6%	13	N-term.	0.74	CYYYCCYYYY	Cys/Tyr	D	1.00
					136	N-term.	0.74	SNNNSSNNNN	Ser/Asn	S	1.00
					156	N-term.	0.78	SSSPSSPSSS	Ser/Pro	D	0.37
					211	C-term.	0.70	NHNNNNHHHH	Asn/His	D	1.00
					240	C-term.	0.71	GGGGGGAAAA	Ala/Gly	S	1.00
KNOPE2.1	34.2-42.0	5.1%	5.4-6.4	5.5%	65	N-term.	0.71	VAAAAAAVVV	Val/Ala	S	1.00
					96	N-term.	0.71	VAAAAVVVVV	Val/Ala	S	0.74
					105	N-term.	0.72	GGRGGGGG-G	Gly/Arg	D	0.75
					115	N-term.	0.70	LSSSSSLLLL	Leu/Ser	D	0.74
					193	KNOX1	0.70	LVVVVVLLLL	Leu/Val	S	1.00
					228	C-term.	0.61	PTTT-PPPPP	Pro/Thr	D	1.00
KNOPE6	37.6-38.6	0.8%	4.8-5.0	1.2%	68	N-term.	0.71	NNHHHHNNNN	Ser/His	D	1.00
					101	KNOX1	0.69	LLPPPPLLL	Leu/Pro	S	0.44
					316	C-term.	0.69	ISIIVIIII	Ile/Ser/Val	D / S	0.01/0.77
KNOPE3	47.1-50.1	1.8%	6.4-6.7	1.6%	128	N-term.	0.68	A-GGAAAAAAA	Ala/Gly	S	0.63
					313	C-term.	0.71	EDDDDDEEEEE	Glu/Asp	S	1.00
					444	C-term.	0.71	SGGGGGSSSSS	Ser/Gly	D	1.00
KNOPE4	40.8-42.0	0.9%	5.8-6.0	0.6%	27	N-term.	0.71	QQQQQPPPQ	Gln/Pro	D	0.30
					47	N-term.	0.71	SSSSSNNNS	Ser/Asn	S	1.00
					153	KNOX2	0.76	QQQQQQPPQ	Gln/Pro	D	0.44
KNOPE7	33.0-33.1	0.0%	6.8-6.9	0.2%	19	N-term.	0.71	GAAAGGGGG	Gly/Ala	S	0.85
KNOPEM	14.9-16.0	2.2%	4.6-4.9	1.4%	71	N-term.	0.65	NNNNKNN-	Asn/Lys	D	0.39
KNOPEM2	15.2-18.1	7.8%	4.0-4.2	1.0%	17	N-term.	0.73	RRRRRRRSSS	Arg/Ser	D	0.76
					79	N-term.	0.71	NKNNNNNKKK	Asn/Lys	D	1.00
					125	KNOX2	0.71	VMVVVMMMMM	Val/Met	S	1.00

1, cv, coefficient of variation, ratio between standard deviation and mean
2, Conservation score as determined by Scorecons server. Scores lower than 0.78 (variable regions) were considered.
3, For a given position (Pos.), amino acid variants within each PRUNOX group were reported.
4, Ref, residues in the *P. persica* members. Alt, alternative amino acids occurring for that position in the other proteins of the same group.
5, Substitution type: similar (S) or dissimilar (D) chemical properties.
6, SIFT tolerance substitution score. Substitutions with score less than 0.05 are predicted to affect protein function.
