# Supplementary material for: Prunus Knotted-like Genes: Genome-Wide Analysis, Transcriptional Response to Cytokinin in Micropropagation, and Rootstock Transformation
Source: Int J Mol Sci. 2023 Feb 3;24(3):3046. doi: 10.3390/ijms24033046 (PMC9918302; doi:10.3390/ijms24033046)
Supplement: Supplementary file 1 [file ijms-24-03046-s001.zip › Supplementary Tables/Table S4. PRUNOX promoters.pdf]

Table S4. Analysis of cis-regulatory cytokinin response motifs on *PRUNOX* genes' promoters of *P. persica* and *P. dulcis*

Legend

	CK binding sites	Type	Name	Ref
	AGATT	Experimental	Core motif (CRM)	[38]
	AGATC	Experimental	Core motif (CRM)	[38]
	GGATT	Experimental	Core motif (CRM)	[38]
	GGATC	Experimental	Core motif (CRM)	[38]
	AAGATTTT	Experimental	Extended core motif (ECRM)	[38]
	AAGATCTT	Experimental	Extended core motif (ECRM)	[38]
	TATATTTA	Computational		[39]
	CATATATA	Computational		[39]
	TATATATA	Computational		[39]

Prunus persica

Prupe.3G212100.1 (STMlike1)

ATCACTTGAACGAAAAAAAATAATAATAAGAGAGAGAAAGAGCAATGGGGTGTCGTGTGCATACTGACTTGGTCTCCCCA
AGAATGCATGTCACAACAAACGCATATAAACGTGTGACTAT**GATCT**T**AATCT**TAGCTTATTATTAGTCGATTATTTCTAC
TTAGAGAGAAGATAATTAAGCGTGGGCAGGAGTCTAAGGCTGAACTGAAATTAATTAAAGACAGAGAGAGAGATACATAT
AGGGCCAGGGAGAGTATTGGAC**AATCC**AAAATCACCAGAGAATATTAAAATGGTTGGAT**AGATT**ATGAAATGCCATCGAT
CACCGAGGTTGACATCTTGATCATTTACCATCAATCATAAACACAGCAGAGTAGGCCACGAGGGGAGCAGATAGTTGGAC
CAAACACCATAGGCCGGCCATT**AATCT**TTCTGCATTAATTGAAACATGGAATGAAAATAACACTTTTTATTATTTTAAAT
TAATAGCACAATAGTATATGAATTAATTTTTTTTTTTACATGTGATTGTGTTATTGGTCAAAAATAATAAGACAGTATCA
TTTTTATTATAAAAACGGTTTTTTTGTCCCTAAATTGTCAGAAATTACCATGCTCTCTCCATTGAGAGAACTAAAAGAAA
AAATGTTTGTTTTGCTTGTGCACTTGTAAACTATTATTAATCAAGTTAATCATTT**AGATT**CACAGTTTTAACTATAAAAA
AAGAACACATTAAGTAAGGACCTTTTTCTCTTTTTAATTATTCTCTTCTTCGAAGTTCCCACTGAGAACTGAAGAGAAAA
AGGCAGTCTCAAACGCTGTCAGTCTGCAGCGCAGCACCAGTGGTAGTACAATAAACGAGGAGATAGATATGTAGATGAAA
ATAATAATATCACAAAGTATAATTAGTTTTGATTATT**AATCT**CATCGGGAAGGAAAGGTTCTCAGGTTTCATTCTGAGCA
AAGGTACACGTTAACTCCGCGTGCACCCTTCTGTTCATTTTTTAAGAACTATAACTCTCTTTTTTCAGTACACTCTACGT
AATTGTGAAATTATACTAGAGTCTAGAGAGAGAGAGAGAGAGAGAGAGAGAGAGTACTGTTTATGTACAGAAAGGAGGAA
AAGAAAAGTAGCCATTTCCGAGGGAGGTCTA**AATCT**ATCAACCCTTTTAATTTACAGA**TATATATA**TATCAGTCATGTAA
ATTTCTGACAGACCTCCACTGTGTCTTTGTCCCCGTGTGTAATTTAGGGGGGAGGCTTGCTTCCTTCTTTCCTTGCTTTC
CTGTACACATAAAGACTGTCACATGTGGAGTTTTAGCTTAAAAACCGGGAGGCAATTCCTCTGCTGAGAAAGAAAGAAAG
AGAAGTTCCACTTCCAGGCCACTGAGAGAGAGAGTGAGAAAGAAAGAAAGAGAGAAGTTCCACTTCCAGGCCACTGAGAG
AGAGAGTGAGAAAGAAAGAAAGAGAGAAGTTCCACTTCCAGGCCACTGAGAGAGAGAGAG**ATG**...

Prupe.4G015200.1 (STMlike2)

AGGGATGAGTACAGGTGACAATTGCACATGACAAAAATGAAATAAGTATGATAAGACTAATATTCTTTATTAAATATTAC
TGTATTTAATTGATGATTATGAACATTGTGAATGTGATGGATATTGGGCTGGGGCTGCCGTTGGTGTTGGGGCGTAGCCG
CGTAGTAGAGAAAGAGAAGAGAAGATAAGGGAAGAAGAGGACAAGGAACAGAGTGCGAATATTAAATGAGTGGAC**AGATT**
GCAATCAAGCTCCAGTAGTAAACAAGAAAAAGCAGCAAAGGGGGTACTGTAGAGAGAGAGAGAGAGAGAGAGAGAGAGAG
AGAGAGTAGCTAGGTAGTAGCAGTAGTGATTCCAATTTCCATGCCCCAAAAAAACCTATGTCTCTCTCTCTCTTCCTCTC
TCTCTCTCTCTCTCTCTCCATCCATCAAAGTTGAAAGTTGAAAGTTGAAAGAAAGAAAGACATACTATTAGCCATTTA
TTTAGGGGTGTGTGTGTCCAGCCGGTTTAGCTCTTCCTCGATTTGATCGATCGTTGGCTGTCTATCTCTCTCATAATTCT
TTGCCAGTAATGGCTGTGCAGCACTGCCACTGCAAGAAAAGGGGGTGAGAGTTTTCATCGGGAAGGTAAAGCTTTGCATC
ACACGACACACAGAGAGAGAAGAGAGAGAGAAAGAAGAGAGAGATACATATATTCACATGACCACTATCTAATACCCTAT
CTATTATCTATCATCAA**TAAATATATATG**AAATGAAACCGGCTGAGTTTCGAGTTTGAGTGAATTACCACCTCCGCCCTT
TCTTTTTCCACCCCCGCGTGC**GGATT**CTGCGTATGAATAAATGACTCTCACAGTCATTCACTTGTGTTTGTGTACAACAA
TACCAATACCCCCCCAAATATTGTATATAAGTAAGA**AGATC**AGAAGTCTGAACTGAAGCCAGCAGTTGTCAAAAGAACAA
AACAAAGAAAGAGAAAGTACTGTTTGTGTGAGTTAATGCATGTGTAGAGAGAGATAAAACCAAGGCATTTTCCAAAGGA**A**
ATCTGAGCAGGCTTTTCTGGCAGACCCCAGCAAATGGAGAAAAACAAACGCTACATTAACTGACCCACTCTCCACCACTA
TCACTCTCACTAACTTA**GGATT**TCCCTGTTAAAAGCCTGCTCAAAGTTTGTAGCATAAAACCCGGGAGAGCCCTCCCTTC
CCCTTCTCCCCTTCTCCTTCCCTTCCCTTGGAGAGATC**AGATC**CATTCATGCACATGTGCATGTACACACCACCCCCCCC
CTTTTCTTTAGCTACCT**AGATC**GACAGCAACCCAAAAAATAAACATTATTGCGTAGGAGAGAGAAAAAGACTTTGAGACA
GAGACTAGTTCTTTGTTTGTGTTTCTTTCTTGGGGTATAATAGCAAAGCCAAGTGAACAAAATAACTTTAGGAAAGTTGT
AAATTGGAAGACAAGATAGCGAGAGAGAGAGAGAGAGAGAGAGAGAGAGAGAGAGAGAGAGA**ATG**...

Prupe.1G416700.1 (KNOPE1)

TGAGAATAATAATATGGGAGTTATTTTTTGATAATTTCTATCCTAAAAAAATGTATTTTTTTAA**AATCT**GATATTTTAAA
GGTTAAAATTATTACAAACAATGAAAAATGTATAATTTGAGAATAACAATGCATAATTAAAATAATAAGAA**AATCT**AATT
TTAAGAAAACCTAAATAATAAAATTCGATTATCTTTAGTTTAAAATAAATATTTAAAAATAAATATGAAATTGAATAATA
TTTGATAAAATTAATATACACTGGTGTAGCAAAATAAAATAATGAAGTGGAACAAGAAAATTGAAAAAATAAAATAAAAT
CAGACTAACAAAAAAAAAAGGAAAAAAAAAACAATCAGACCAATGCACTGGGTAGAAAATGATAATTTGTGGTTTTCAAT
TTTTCTACATGAGTTTTAATTTGTTTTTAGAAAATGAATTTCAGTAAAAATGAAAAGAACATACCGAAATCATTTTTGAT
AGAAAAAAAAAACGGAACTTTAAAAACGTTACCAAATGGTCCCTTAGTCACTATTTTGTTAGAATATAGTATCTTGAAAG
TTGTCCATGAATATTTTCATAATTTTAGTAATAGTAACTTGTTG**AATCT**ACAATTCAATTAACTAGTATAATTTTTTTTT
TGTAGTGTCACATGAAAAAATTATCTCATGTTTTATTGCGCTATTGGTCGGGAATAGCAAAAAATGCAATACCCGTTGCA
TGAAAGTCTTAGTGGAGCTATACTAACTTAAATTTTAAAAAATTAAATAACAAAAAAAAAGAGTATTTTTTTATAATAA**A**
GATTCAAAAGAAAAGAAGAAAGTTAAAATAGTGCCCCAAATGTC**TATATATG**CAAAAAGAAACGAAAATCATATTAAATT
CAATTAAACGAGAAAGGAGCAAATAAA**AGATT**CAAAAATGGGAAACGTCCGTGAAACAAAGGTTAAAAATCAAGACTAAG
AGGGAGAGAGCAAGA**AGATC**AGAGAGAGAGATGGAAGAAA**AATCT**AC**AATCT**ATTGAGTTGCCTTATCGCTAGGTCTCCT
AGAGAGTCATAGCTTTTAAGCATTTTCAAATATTTCTCTTTTCTTTCAACAAAGCAAAGCCAGTCTCTTTCAACCTCACC
CTCACAACGGGGAGCCCCTAGTTGCCGGTTCCGGCCACGCTATCTCCTCCACTTCCCTCCTCTCTCTGATATTCCCAAAC
CATCACTCATGATTCAAACGCAAGCACAATTGATACATATTCTGTAACGCAATTATAACATGCTTAGTTCCCCGTTTAGC
TTCACTTCCTGGCCTCCATAATTTCATGGCTTGCCAGTAATATTACTTCCCATTTCTCCCACTTAACTCCTTGACCTTTT
TTGCGTATAAAAACTCCGGCCATGCTTTGTTTGTCCACTAGGGTTTTGGTCTCTTCCTCTCTTGCTCTATTTCTTCTTCT
TGCTTTAGGGTTTTAGTTTGCTCTTGTTGATTGGAAATCATCTGGGTTTATTGAGAGGAA**ATG**…

Prupe.1G249600.1 **(KNOPE2)**

TTATCTTCTTCCAACTCCCCACCGTCCCATAATATAAGGGTAGGGTTTCAAAAAGTCGTGAAGACGTTTACCCTAAACGC
ACCGCATAATGAATTGTTCTACCACCACCGACGTCCATGTTTTCTATTGATTTTCCTTTTCAAGTTCGATTTCCATCATA
TTAAAGCAACCAATTGAATTTGCTCCCAAAATGTCAACTACACACA**CATATATATA**TATATATTCATGCATGCGTATACA
ATATACAAAATTAATATTAATTAATGCAGATGCTGGTGGGTTCCAGTTCCATATGCATCTCGAGCTCATTGGGGAGGAAT
GCGTGCCCTCCACGTGCTAGTGTGTCCCACGTGTAGTGGCCGCCTGTAGGTATGCATGGCCCACCACTAGAATTCGTCTT
GACTACAT**AGATC**ATACTTTTTATCGAATAATTTTTCCTTTGTCGAATGCATCAATTATCAACATTATCTACCTACAACT
GTACTGCAAGTTATTATTAACTCCCCAGTAAAAATATAAAAGGTAAAACAG**TATATATA**GAAAAGTTAACAGTAAACTTT
AATAGAGCCACAAATAGGGTTATACAATGTGAATTGTCAATTACTAAAATTGGGCAGAGTCGTCAGCATAAGAAGCATAG
GTATCATTGTACAATGAGTTATATACACAATTTATACAATATTATT**TATATATG**GAAAAGGAAAAGTCTCATCTTGCCAT
TTACAAATACAAAGGCTGCTTTTTATATTTTATGTTTTTTCTGCTGGAATTTTAAAACCTACTTTGCTCGTGCCTCGAGC
CTCCTCTTTCTTTTTTTCCATGTTTGGTCAATTTAGGTATTGCATCTTTCTAATTTTGTTTTGGTGTATCTAGCTGTCAG
TTTGGGAGTGTTTATAAAA**GGATT**AAAAGTGTTTTTGACAACAAAAAAATATTTCTGACAACTATAATTTAGAAGTACT
TCTAGGTCATAAAAGCACTTTCTAGAGTATACTTCTTCAAAAAGT**GATCT**CAAATATTTTTTCTTATGAAAATTTATAAT
AAAATCATTTACGATCGCTAGAAGTGCTTATGAACAAAAATGCCTCATAAAGAAGTAATTGCAAACAAGCATTAAACATT
GTTTACACCTTGAAAGTAACACACACGCTAAATAA**TATATATA**TTTTAAGTAACAACACAGTAGAAAAGGAAAAAGATGA
AAAAGGAAGGGGCTATTACATATCTAATCATATATCACTTAGTTGTTAGTTGGACAACAAGCACACTCCCCAAGAACACT
TCCAGTTACCCTCTGATACTCCACTTCCAGCAAATGTTCTCTCAGTTAAAAATTTTGTGGTTAAAATTTCAAGATGAATA
GAAATAAACAAATATACTGCCTTTTAGTTTATTTTATTTTATTTTTCTCCTATAGCTGTAGCTAGAACTAGAAGAAGACA
TATAGAAAGAAAGAAAGAAGAAGAAGAAGATATACTTTTGTTGTCTGCCATTTGAATTGG**ATG**…

Prupe.5G171100.1 (KNOPE2.1)

AACGTTATGTCATATTTGTGTTCATGCCAAGTAATGTTCAAGATGAACATGTTAATTCGAGTCAGTGGCGGAGCCAATGC
ATGCCCAGGGAGGGCATGTGCCCCTCCTCAAATATTATTGTATGGATATTTAGTTATAGTAATATTATACAAATATTAAG
AAATTCCTCTTAAATTCTTAAATTTCCCTTTTTTTAAGTTCTCTATTTTATTCTCAAGTCTAAATTTCTTTTGAGTTGTA
TATATTTTATTATTATGAATGAATGTTGGGTTATTTATACAAAAAGTAACAAACAGAGAAAGTAAAAGGAGTTTGAATTT
TTAAATGCGCCCCTTCTCACTTAAATTTATGACTCCGCCACTAATTCGAGTCATTTTCTTTTGCAACTTGAACTATTACT
CTTGATGTATCATTGATCATCTAACTCATTTCGTACACACCATTCATTGACATTACAAAAGAAGAAAAAATTTCAATATA
AAAAATGTCAAATTTCGTTCACACCAATTATTGGTTGTATTGTAT**GATCC**ATTGGTATTTATGTACTGGTGCAATGCAAG
GCAAGA**CATATATATATTTA**TATGTTAAAAAAATGTAATAAAAT**AGATC**ATGAAAATAAATAGTGTAGATGGGTCATGCC
ATGCAATGGGTTGAGAATTGAGATGGCCACTTTTTGTAGTTTAGTCGAGACTCGAAAGGGAAGTGACTTCGACGCTCTGT
GCAA**AGATT**TGGACCTAGTCATGATCAGACAACTGAAAAGAAGTTCAGCTC**AGATC**AACAGGGCCAAGGCTCCAGTTTCT
CCTTGTCCGTACGCCAAAATTTTACACTTCCCAATAGACCATCGCTT**CATATATA**CAGTAA**GGATC**AATTTCTTCTTGCA
AATAAATTCCTGTCTATAATTAGTTTCAATTTTTTTATGAGTTAATTTTG**CATATATA**TTAGACCGATAAAGTAATATTT
AGTAATTAATTATATGCTAAT**TATATTTA**CCGTATACATAGAGAATGAAAATAAATGCCAAAT**AGATT**TTAAAAAAAAAA
AAAATTATATAACAAAGAATTTATAACTCAAATAATTAACAACAGTTATTCATGCATTCGAAATTCTGTGTTGG**AATCC**C
TCCTCCAACCCTATATCGTTTGCATGAAAAAAAATATATAATTTGAATTTGGCTCAATTATGTTACTTTACACGATAAAG
ATAGGTGACAACAAACTATTTTTGGGAAGCATTTCTCCGAGCATGGAATACTGGTTGTTGACCTTTTGACTTGATGTACA
ACTTCTACATATACATACAGCCCATTACCTACTTATAGTATAATTAATTAAACAATTAATGTGTGCGCTCTCAATGGCCT
CACACTTCCACTTTCCATGTCTATAATCACAAGCCTAGCTCTGGTCTTTTTGTCTTTCCCTAAGGCTAAGCAGATATATA
GCTAGCTCACCCTCTTCTCCTCCTCTCCTCCTCCTCCTCCTCCTCTTTGGAAAGCCAACA**ATG**…

Prupe.6G312900.1 (KNOPE6)

TAATTACAATTAATAAAATCACGCTCGAATAAGACATAACGTAGATACGATGAATTTATAAAGTTGTCTTTTGATGTATG
TAAGTTTTACCATTATATTCATATACATGAACAGTTAAC**AATCT**TCTACGTCCTAAGTTGATCACTCTCGAAAATAAAAT
TATAGATAATTGGCTTTAGCTAACAAATGTT**AGATT**GTTTGGCTAGTAAATTAAGCTTAAAG**GGATCT**TTGAGCCCAGCT
GAACAAATGCGAAAATAGTCACCCCTCCTTTTGGCTTCAATTATTAGAGT**GGATT**GCACTATCTGCTTCTCTCAATTTAC
TGGGAGGAATATTCATGCGGAATAGTTAAAGCTTTGGCACTTATTAATTTATCAACAACCAAGAAACAAGAGAAAACAAA
GTTAGGTTGAATACAAGGAAATGTTACGGAAGTGGAAAAAGAATAAACTAAAAAGAAAATAAACCATGTCATCAATTTTA
ATTTTAGGTATTCGCTCATGCGATCACATTTCTTCATCATCTGTCACATCTCTCATCCTTGTTTCTTTTCTATCATACAT
TTTCCCTTCTGTCTGTCTTTGGAGCCAAAGAACAAAAAAGCAAAACTGAAAGCCAAATAAAATGCTACCAAACAAACCGT
GCACTGGTGTAAACTGTTCACTTCATTGATGTGAGAGAGAAAGGTAACATGCAGACACTAATTATTTTCAGACTAATCAT
ATTTTTTACCTGGGTTACTGGTTTTTTAAACTTAATTCATTCATGTG**AGATT**AAAAGTGAGAGTAGGGTAATTCAATTT**A**
ATCCTCCAATTATCTGGTTCTTGTTCCCAACCACAACCAGGGCCTGTGGCGGTGAAAAGAACAGCAACATGAACAGGAAA
TGGCTGATGAGTGAGTAAGTACAAGCAAAGTCTCCTGAAACCACAGCGTTGATGTTGTTGGGCCCCAGTGTTGCAGTCCC
AGAACCAATAGGGTTTTGAAACGTAGTAGAATGTCACGTCACAGTTTAAAGGCCATTCCAAAGTCCCAATGCTCTGAGGA
AGGTGACGGGTGGACCTCTATAAATACGGGCTACGCCTCTCTCTCCCTCTAACTTCAAATCAGACCTTTGTTTGTTGGTC
ACCGTTTCAGTACAGCCTTTTATCCATAGTGC**AATCC**ATTTATCTCTCACACGCGACCCCAAACACAAACAGTTTCCTCA
TGTTCCTTCTCCACTTTCTCTCTGTGTCTTCTCTTTTCCTCAGGTTTCCTTGGAAAAGAAAGTAAAGTCTGGTCCTTTTC
CATGTGAGAAAGAAAGCCCACAAAACAAAAAAACAAATGCCACTTTGGCTCCTAGTCAAGCTAAAACCCTTTTCTTTTCT
CTCTCTCTGTGTCTCTGTGTCTCTCTCTCTCTCTCTCTCTCACTCACGAGCTCATTCTCCACTGTAGAAGTATTATTGTT
GCTTAATTAGCTACTTCTTTTTCTTGGACATTAGAGCTTGAATTCGAGAAAGATATATGT**ATG**…

Prupe.1G486200.1 (KNOPE3)

TTACCGGTCACAATTTTT**GGATT**AAGCTGCATTTACTTAGAGTATTATTTCGTGTTTAAGCTCCGTTCTAATTGTAATAAC
TATTTGATTATGTTAAACCTTTTTTTTTTTTTTTGTAA**GGATT**AATATTCTGTATCATTCTAGACAAATATCTAATTCAA
TTCTAATAGTTCATCTAATTTCAAATCGCATACGATTTATGGATGTTTAAAAAGTCTACGCAAATGGACAAAACTTTATC
ATCTTTATTTATTGGGGTTTCATACAAAAGTAAACATCATATCAGTGATTATTAAATT**AATCC**CCCATCAACGATAATAA
TATGCC**AAGATTTT**CATTGAGTTATTTTACTTTTTGTTCTTTTTTTCCTATGCTAACTTCTGGTTGTGAATTTTTTTTTA
AATTAAAAAATAAAGTAATGCTATACTTAACACATTTTTATCTCATATTTTTGTACTAAATGATGTGGCATATTCATATCA
TATGTCACATTAATTAAATCAATAAAATATATTTTAATAAATGAATAAAAAGTATTAATCATATTAATTAACTGATGTGG
CATGTACATATCAGCTGCCACATCATGTAATATAAAAATGTGGTAAGACTAGTATTATTCTCATAAAGTGAAAGGT**GGAT**
TGATAATAACTGCCCGAATAGTCCGTTAAAAAAAAAAAACCTACCCGAATAGTA**AATCT**CATATATTATGTTAAATGGGG
GAAAAAAATATAATAAGTGGTCTTATAAAATTTCACTCAATCAAACCCAAACATTTTGCTTCTTAATTAAAGA**AATCT**TT
GCTACCCTTAATT**AGATT**CAAAATACCATTATTTTTCTTTGTAAAGAAATTAAAATACCATTATATTCTAAAATCAACTA
TTT**GATCC**GAATTTTCTATTCTTTCAACTCCTATATCATATTGTGAAATAATAATAATAAAATATTCGTTTAATAGTAAT
AGGGGACCCTTGGGCTCCTTGGCTCCTTAATTAATATTAAAACCTGCCACAA**AATCC**CACCATTTTTTTTCTTGCTAAAG
CAGTTTCTCGAGCTCATTTTGCTTGCCTGAAAACCACTCTGTCCAAAAACCTCTCTCTGTCTAAACAGCTCCTCAATTTC
CTCATATACGTTTTTGGTACCATCC**CATATATA**AGCTCTCAAAGCTTTAACTAAACCCTTTCAATATCTCTCTCCCCAAA
ACTCAAAAAAGCTCGCATCTCTCTCTCTCTCTCTCTCTCTCTCTCTCTCTCTCTAAGTTTCTACACACACACCGTGTAGTTT
CTCTACACAGAGAGATAGATATAATCA**AATCC**AGCCATATATCTTTGTCTTGTCTCTCTCTCCTATCTTCTCAGACTCTC
AGAAAGCTTCTAAATTTTCATATCCATGTCCTCAGAATCATCTTCTACAATTGCTCCTTTTTAATTTGTAAAACACAGAG
AAACAGAGTGTCCTCTGTCTCTGTCTCTGCAATTACTTTAATACTCAGCTGATCGTCGATC**ATG**…

Prupe.7G254700.1 (KNOPE4)

CTTTGATCATTATGTATTAATCACGATTATTTCTTACAAGTCT**AGATT**GATGTGAGAATGCTAAAGATGACCAGTTCTTT
TC**AATCT**TTATAAGTCGAGTATAGTGGTTACCGTGGAGGGTGCACGGGGTCATTGGGTAATGCTCAATAGGCGTTCGTTT
GATATGTGGATATCAAGTCATGAATTGGATATACTAGAATGAAAGTGTATGTTACGA**AGATT**ATGTATCATGTTTTTGGT
GACTTTCTCATATTTAGCACAATTTGGTTTGAATTTCAAGTATGCATGTGTTAAGAGTCATTTAAAAGTAATAAAAAAAA
ACTCGAATAATTTTTTATTTCTTAGATGTAAGTACAATATAGACAATAAAAAATTATAATCATTTAAATATGTATGTATT
AGTAAATGCAGATAAATAAAAAAATAAAATGATTCCGATACCAATTATTGATAAATAAGCATGTCATAAATAACAAGTAA
ACATACCATAGAAGGAAGGTAAGAA**GGATT**AAATGATGCGCGCTTATTGAACATCATAAGGTATTGCTTTCTTGTCTATA
T**AGATT**TGTTATGAGTGAATATTTTATTGAGCGGTGTCCTGCTATTTTGTCTTTGTCTTGTAAAGCTATGTTGTTTGAGA
GCGTAACCCAAGGGTTAAAGGAGAAAAGTTACTTCTGACAAAATTAAAAATAAAGGAGAAAAGTTGCTGCGTCCTGCCTA
CTTTCCTTCCACTTTTCCTTCCTCTCTCTCAAGACTCAACCATTTAAAAATAATATAAA**GGATT**TCAATGTCGACTGCTG
TGCTGTCCTTGGTGCAGATGAAGGGGGTGGAAACTTGTCTCAAAATCATGTAAGAAACTTCTTGCAACACTACAAAAACC
TAACTCATACAA**AATCT**ATGT**TATATATA**TATATTATATGCCTCCAAATATTGGTACGTCAGATATCCCAAGGGGTCAAG
TATGGAGCAATTAACAATCACAACGACAATCACGCCCCAAAATGCCACCCAACGGTCCAAAACATTTCAATAATTTTTCC
ATCA**AATCT**CACCGTTGGATGCGTTCTGAGCTTTCTATACATCAATGGCTACTTACTATTACAAGTCTACAACACATCAC
AATTTTCAATCATCAATAAATCAATGTTTGTCTTATTTACTGGGAACGGCAACTCCCAATTACATCGCCACATGGCACTC
TATCTCACCGTCAAAACATCCTCCTCGGTCCTGGCGTCACCCATCCATCACTCAACAAAGTCGTCGTCTCCCAAAAAAAG
AGCTTCTGCGCTCCTTTAATTGCTT**AATCC**AGCATTCAAGTTTCAAACTTTTAACAGCACCACTTTCTCTCTAACTCAGA
ACATCACAATTTTTTTTTTCCTCTCTCTCTCTCTTTCTCTCAGCTCCAAAAACTGCTTTTTTCCGAACCACGTTTTCTTG
TATGTACTGAAACTAAAAGCTCTGTGCTTTTTTTTACAATTTAATTGCAAAGACCAAAGTA**ATG**…

Prupe.5G088100.1 (KNOPE7)

AAATATTTGAGAAAATGTGAAAAATAATATTAATAATAATAATCAAATTGATATTGAAAAGCATAATACACCAGCCAAAG
GAGACGGCAACAGTGACAGCTCAACCCTCAGCCGACTTTCTTAAATAACAAAACATATAGAAAATGTAAGGGTTTGGTAC
CAAAAATACGGTGCCAATATTTTGATTAATGGAGCACCCTACCTATGTAGTTGGGTGACTATTACGTTGACGTATTTGTG
GTAATTCACAATGTATTATAACAGTTAAGAATTACCTACATACGCGTTGATAAAATGATATTTTAATTCTATTTATTGTT
ATATTACAATTAGTCGTCAACACGTACATAAATGAGTCATAACCGTTAATGCATGTATGATAATTTTACACTATGATGAT
GAATGCTTACATTTTAACATGGATAGCATATATGGCTGGAATTGAGTATCATATATTGATATGCTTATTTATTATTTATC
CACCCTGACCCTAAAAAAGTTAATAAAGTAATTGTTACACAACTTTATACAGTGTGACCACAAGAGCTATAGCTAGAAAG
CTACATATATGTAGATAGACTGTAGTGTTAGTCAACAATGAATTCATATTGAAAGGAGTGCTCCATTTATGAGACTGTGA
AGTGTCCAAATCAGGTCTTTAAATAGATGGTTGAAGTCTAATTTGTGATTTTAAAAGGGAAAAAAG**AATCC**ATATTTTCT
TGCTGATAATTAACCCAAGAATTATTGCATACCTAATCAAAATTCATTCCTGATATACCA**AGATT**AGTTAGAATTAAATA
CTTAACCTCCCCAAGAAAAACCCTTTTTCTCTGAGTAAGCTTTAAGTTGAATTAGGACCCCAGAAAAACAAAATCAGAGG
AAATTATAAAAACAAGAGGAAACAGAAAAGAAATAAGCAATCAAATTGTCAGGCCACAGAAGCAAGCAGCTTTATCTTGT
TTCTTTTTATGAGCTCCCTTCCAACCGCTTTCATGGTAATGATTCATTCTATTGCCCCCTGTTTCTATTTTCATCCTCAG
TCAGTCCCTCTCTAAGCTAAGAACTCATTTCTCAAGAGGCATCAAAACCATTGAAAACCTCACAAAAAGCTTTCAAATTT
TCCTTTCTCTATATCTGTGTATGT**AAAATCTT**TGTTTTTCTCAGACGTGGTGCAGAAAGTTGTGTGTCTC**AGATCC**CGAT
ATACGGCTCTCCATGGCATCTTTCTTACACCATTAACGATAAAACTGTCAAAGCTATCAAGTTTGCAACTTTCTCTCCTT
CTCTCTCTCCCTTTCCCTCTCTCTCTCTCTCTCTCTCTCTCTCTCCCACACTGGCATACACCATAATGGCATAAA**AAT**
CCATTCTCAAACACAAGGGGTCTCTACTTTCTTTGTGGTCCTCAAAACCCAACCCTCACATACCCTTTTGAAATCACAGC
TTCCTCTACAAAACCCATTTCTCCAAATCACAAACTGGTTTAAATTTGGCCCTCTAGAAA**ATG**...

Prupe.5G236700.1 (KNOPEM)

ACCAT**AGATT**ATAGGGTACC**AGATT**AAGTTAAAATGTGTTATAACTTGAGGAGAGAGATAAAAAAGGAGGGTCGGGGCAT
GAAAATTAATTATCTCTTAGGGCATTTCTATTTATTTGCCATGGTAAATGATAAGGGGAAGCAATAGCTGTTATCATTTA
CATGAATAGTGCTTGCCCCAGCAATTTTTTTTTTTTTTGGTTTTCCACCAAGTCTTGCCTTTGGACTCGCCCGGGCTAGTG
GAGCCCAGCTCTTGCCCTAGCT**AGATT**TGGGCTTTGGAAACC**AGATT**GGGGGCCTCTTGCCCGGGCTAGGGCCTCCCGGT
TGAGGTGGGCAGCCAATCATATCCAAGGTTGCTTCATTCAGTTAGTTTCAGAGAAAATTTGTTAAATCAGCTTATGTGG
AAGCAATGTGACATTTCAAGTTGGGGTATATGGTCATTTCGCATATACTCACCCATCAGTTGTATTTAGCCCGTCAATTT
GCCCCAATTCTTCCCAAAATCAAAACCCTAATTTGAGACCTGCAACAAAGCCACTGGAGAACTCTAAATTGGCGTGTGCA
ATAACCAAATGATGTG**AATCC**TAAAACCAGTC**GGATCT**TGTGTTAAAATAA**AGATT**ATGAACAACTTTAGTTTGTTTGTA
TGATATATAT**TATATATA**AAGGAAAATGGTTGAATAATAATTTATTAAAAAGCTTTTGGT**GGATC**AATTAATGTCTGGGT
ACGTACGTACAT**AATCT**ATGATAC**CATATATA**TCACAGGCACAGCAATGAAGGGTCATTTTCGTATGAGTGCATCCACAG
TAGTGGAAACATTCTCTTCCTCAAATAAATGTGAGCAAACATTGATAAAAGAAAGAAGCCCTGATACATCAACAGTGGGG
AATTTGGAAGTACTGCTTGAAAAAGTGAG**GGATT**GACTAGTAAAAAAGGAAACTTATATAAAGACACACAATGCAGACGG
ATGAATAGGAAGAAATGTACTAGCAAAGTCCCCGCTGTTCGAGAAACCCCTTTGATATGTTACTTTCTTTCATGTATAAA
GACAGAAAAAGAGAGAGAGAGAAAGAGAGAAAGAGAGAAAGAGAGAAAGAGAGAGGGAGAG**AGATT**TCAAAGGCCAAAAA
CCCACACCCCAATTACGCATTAGTGTTTCTGACCGACTGATCATGATGAGAGACTGATCAAATTAATATATGAAATATAA
ATTATATGCATGCATGAGTCATTCCAGTGGTGAATTCCAAAAATGGGAGCGTCTCCGTACTCTCTTTGCACTGATCACTT
AGTTGTAATATATAATAGAGAGAGAGAGAGAGAGAGTCACAGTACTAGTAGACTAG**TATATATA**TAGTTAATGTGAACAA
AAATGGTAAGACTGACTTGTATGATGCATAATTGTTGTTAGTGCAGTCCAGAGAATTTGTATTTGCTTATACTTATAGG**T**
ATATATAGCTAGAGTC**GATCT**CCTCTCTCCAGGGTACGTTGGA**AGATT**TGTAATTATAAT**ATG**...

Prupe.1G335000.1 (KNOPEM2)

AT**GATCT**GCTGCATATGTAATTATCCAAGTTTAGTTGTCTCTCTATCTCTACCAAAAAAAAGAAGAAGAGTATTTTCTGG
ACATTTTTTATTATAGTTTTTTCCTTGGGTGACTTTGAAAAGCATGATTCTATTTCTATGTA**CATATA**TATATATGACAT
AATTGAAACTCCTCCAAATCGTTGCCTTTTATTAAATTTAAATTTGTACCCTCTTCCGTTTACCAAATTTTGATTGCTCA
GCGGTGGTAGAATC**GATCC**ATGTAGTTGATATT**AGATT**TTGACTTGCCAATGTATACAACAAAAGTACTTTTTTTTATAA
TGGTTTGTAGTTGTTTATAAACAACGATGTTGTGGTGTCACTTTAATTTTGTG**AGATT**TTA**GGATC**ATTTAATTCTATAC
GTAAAATTAGAGTTAAAAATGTAAAATCGAAGCAAAAGACTCATTAGTGTAGTAGTTTGGTATATTACCACCTCAAGAAA
ATATTCATGCACTTGAGGTTTTGTTCG**AATCT**TTCTTTTCCTAATATTAGTTGAATTTAAAAAAATAAAAAATAAAAAAA
AGAATAGATAACAATGAGGATAAGATAAAATATAAATTTGAGAGGGTGAAGATACTTCACCCATTAATTTCT**CATATATA**
TATATATATATATATATACATACACTGTGCACACATCCGAAAGAACAAATAATTTGCATGTTT**CATATATA**ACGATTTCA
TGTGCTACTCGGTGTTTCT**AGATT**GG**AATCC**AA**AATCT**GCATAAATTCAATCAACAAAAGGAAACGAGTGCTCATAACTT
GTTTGGTTGTTTCCGCAATGAATGCGTGCAGTTTGAATTAAGCTCCCAACTAAACCACACAAGAACATGTATGATGCATA
AACTAGCTATTAATTTGTAACGGTCCTTTCGTCTCTGAT**GATCT**CATATATTTGTATTTGTATGTATCCCTCTCTTGTGT
TTCTGTTTGGTCAATGAGTGACGCAACAAAGTAGAAAACTCCAAGTTGGTCAATGAATGACGCAAAAAAGAAAAAGAAAA
AGAAAAATTATTGTCCAAATGTAAATGAGGGAAAAAGAAAAAGACAAAAAAATATAGAACTTGCTTTGGTCTTTGGGTGA
AGCAAAGTACAAAGTGAACATCAAGTATGTTGCGAGACAGACAGCTTCGTCTTTTATCGAGGAGGAGAGAAATGAAACGT
CCATAGGTTTCTTTCTCGGCATAAAAGATGAAGCAGAGAAACTATTTGATCAAGTCAGTAGTCGTCACTCTCCCAGCTTT
TATCGTGATTTATGCATCATGCACATATCTACATACTGATTTGCTTCACATATTTTTAAACTAGCAAAAACAACTACTGT
TCATATTTGTTTTAGTACCCATAATTATTATTAATTATTAACACACCTACAAGGGGTGGCTACTTATCTCATCATCATCT
TCTATGTGGTGTGTTTGAATTTGAGAG**AGATT**TCAGAGAGAGAGAGAGCTGGTGAGAGAG**ATG**...

Prunus dulcis

Prudul26A011470T1 (STM1-like)

TCTCCCCTCCACAGCTCCCCAAAGCCCACTTCCTTTTGTTCTTAAAATTATTATGAATTAGACCCATACGATATGACTGC
TCCTGACGAAG**GGATT**TAGTTTTCTTCTTCACTTGAATAGAAAAAATAATAATAATGAGAGAGAGAGAGAGGAATGCGGT
GTCGTGTGCATACCGACTTGGTCTCCCCAAGAATGCATGTCACAACAAATGCATATAAACGTGTGACTAT**GATCT**T**AATC**
TTAGCTTATTATTAGT**GGATT**ATTTCTACTTAGAGAGAAGATAATTAAGTCGTGGGCAGGAGTCTAAGGCTGAACTGAAA
TTAATTAAAGACAGAGAGAGAGAGAGAGAGAGAGAGAGAGAGAGAGAGATACATATAGCGCCAGGGAGAGTATTGGAC**AATC**
CAAAATCACCAGAGAATATTAAAATGGTTGGAT**AGATT**ATGAAATGCCATCGATCACCAAGATGGACGGTTGACATCTTG
ATCATTTACCATCAATCATAAACACAGCAGAGTAGGCCACGAGGGGAGCAGATAGTTGGACCAAACACCATAGGCCGGCC
ATT**AATCT**TTCTGCATTAATTGAAACATGCAATGAAAATAACACTTTTTACTATTTT**AGATC**AATAACACAAGAATATAT
GAACTAATTTTTTTTACGTGTGATTGTGATATTGGTCAAAAATAACAAAACAGTGTCATTTTCATTATAAAAACGTTTTT
TTTCCTAAATTGTCAGAAATTACCATGCTCTCTCTATTGAGAGAACTAAAAGAAAAAATGTTTGTTTTGCTTGTGCATTT
GTAAACTATTATTAATCAAGTTAATCATTT**AGATT**TACAGTTTTAACTATAAAAAAAGAACACATTAATTAAGGGCCTTT
TTCTCTTTTTAATTATTCTCTTCTTCCAAGTTCCCACTGAGGACTGAAGAGAAAAAGGCAGTCTCAAACGCTGCCAGTCT
GCAGCGCAGCACCAGTGGTAGTACAATAAACGAGGAGATAGATATGTAGATGAAAATAATAATATCACAAAGTATAATTA
GTTTTGATTATT**AATCT**CATCGGGAAGGAAAGGTTCTATGGTTTCATTCTGAGCAAAGGTACACGTTAACTCCGCGTGCA
CCCTTCTGTTCATTTTTTAAGAACTATAACTCTCTCTTTTTTAAGTACACTCTACGTAATTGTGAAATTATACTAGAGTC
TAGAGAGAGAGAGAGAGAGTACTGTTTATGTACAGAAAGGAGGAAAAGAAAAGTAGCCATTTCCGAGGGAGGTCTA**AATC**
TATCAACTCTTTTAATTTACAGA**TATATATA**TATATATCAGTCATGTAAATTTCTGACAGACCTCCACTGTGTCTTTGTC
CGTGTGTGTAATTTAGAGGGGAGGCTTGCTTCCTTCTTTCCTTGCTTTCCTGTACACATAAAGACTGTCACATGTGAGTT
TTAGCTTAAAAACCGGGAGGCAATTCCTCTGCTGAGAAAGAAAGAAAGAGAGAGAGAGAG**ATG**...

Prudul26A016643T1 (STM2-like)

CCTTCCTTTATAAGAACAAGTGTCCAAGTATGGAGAAAAGATAAATAGCAAACATATATTCTTGACGCATTTGAGTGTCC
GATAGGGATGAGTACAGGTGACAATTGCACATGATAAAAATGAAATAAGTATGATAAGACTAATATTCTTTATTAAATAT
TACTGTATTTAATTGATGATTATGAACATTGTGAATGTGATGGATATTGGGCTGGGGCTGCCGTTGGTGTTGGGGCGTAG
CCGCGTAGTAGAGAAGAGAAGAGAAGATAAGAGAAGAAGAGGACAAGGAACAGAGTGCGAATATTAAATGAGTGGAC**AGA**
TTGCAATCAAGCTCCAGTAGTAAACAAGAAAAAGCAGCAAAGGGGGTACTGTAGAGAGAGAGAGAGAGAGAGAGTAGCTA
GGTAGTAGCAGTAGTGATTCCAATTTCCATGCCCCAAAAAAACCTATGTCTCTCTCTCTCTCTCTCTCTCCATCCATC
AAAGTTGAAAGTTGAAAGTTGAAAGAAAGAAAGACATACTATTAGCCATTTATTTAGGGGTGTGTGTGTCCAGCCGGTTT
AGCTCTTCCTCGATTTGATCGATCGTTGGCTGTCTCTCTCTCTCATAATTCTTTGCCAGTAATGGCAGTGCAGCACTGCC
ACTGCAAGAAAAGAGGGTGAGAGTTTTCATCGGGAAGGTAAAGCTTTGCATCACACGACACACAGAGAGAGAAGAGAGAG
ATGCATATATTCACATGAGCACTATCTAATACCCTATCTATTATCTATCATCAA**TAAATATATATG**AAATGAAACCGGCT
GAGTTTCGAGTTTGAGTGAATTACCACCTCCGCCCTTTCTTTTTCCACCCCCGCGTGC**GGATT**CTGCGTATGAATAAATG
ACTCACAGTCATTCACTTGTGTTTGTGTACAACAATACCAATACCCCCCCAAATATTGTATATAAGTTAAGA**AGATC**AGA
AGTCTGAACTGAAGCCAGCAGTTGTCAAAAGAACAAAACAAAGAAAGAGAAAGTACTGTTTGTGTGAGTTAATGCATGTG
TAGAGAGAGATAAAACCAAGGCATTTTCCAAAGGA**AATCT**GAGCAGGCTTTTCTGGCAGACCCCAGCAAATGGAGAAAAA
CAAACGCTACATTAACTGACCCACTCTCCACCACTATCACTCTCACTAACTTA**GGATT**TCCCTGTTAAAAGCCTGCTCTA
AGTTTGTAGCATAAAACCCGGGAGAGCCCCCCCTTCCCCTTCTCCCCTTCTCCTTCCCTTCCCTTGGAG**AGATC**ATATCC
ATTCATGCACATGTGCATGTACTCACCCCCCCTTCTTTAGCTACCT**AGATC**GACAGCAACCCAAAAAATAAACATTATTG
CGTAGGAGAGAGAAAAAGACTTTGAGACAGAGACTAGTTCTTTGTTTGTGTTTCTTTCTTGGGGTATAGCAAAGCCAAGT
GAACAAAATAACTTTAGGAAAGTTGTAAATTGAAAGACAAGATAGCAAGAGAGAGAGAGA**ATG**...

Prudul26A000871T1 (KNOPE1-like)

TGTACGACAATTTGAGAATAATAACATGGGAGTTATTTTTTGATAATTTCTGTCCTAAAAAATGTATTTTTTTTTTTAA**AA**
TCTGATATTTTAAAGGTTAAAATTATTACAAACAATGAAAAATGTATCATTTGAGAATAATAGTGCATAATTAAAATAAT
AAGAA**AATCT**AATTTTAAGAAAACCTAAATAATAAAATTCGATTATCTTTAGTTTAAAATAAATATGAAATTGAATAATA
TTTGATAAAATTAATATACACTGGTGTAGCAAAATAAAATAATGAAGTGGAACAAGAAAATTGAAAAATTAAAATAAAAT
CAGACCAACAAAAAAAAAAAGGAAAACAAAAAACAATC**AGATC**AATACACCGGGTAGAAAATGATAATTTGTTGTTTCCA
ATTTTTCTACATAAAGTTTGAATTTGTTTTTAGAAAATGAATTTCAGTAAAAATGAAAAAGACATACCGAAATCGTTTTT
GATAGAAAATAAAAACGAGGCTTTAAAAACGTTAGTAAATGGTCCCTTAGTCACTATTTTGTTAGAATATAGCATCTTGA
AGGTTGTCCATGAATATTTTCATAATTTTAGTAATAGTAACTTGTTG**AATCT**ACAATTCAATTAACTAGTAGAATTTTTT
TTTGTAATGTCACATGAAAAAATTATCTCACGTGTTATTGCGCTATTGGTCGGGAATAGCAAAAAATGCAATACCCATTG
CATGAAAGTCTCGGCGGAGCTATACTAACTTAAATTTTAAAAATTAAATAACAAAAAAAAAAAGTAATTTTTTATAATAA**A**
GATTCAAAAGAAAAGAAGAAAGTTAAAATAGTGCCCCAAATGTC**TATATATG**CAAAAAGAAACGAAAACCATATTAAATT
CAATTAAACGAGAAAGGAGCAAATAAAAGAGTCAAAAATGGGAAACGTCCGTGAAACAAAGGTTAAAAATCAAGACTAAG
AGGGAGAGAGCAAGA**AGATC**AGAGAGAGAGATGGAAGAAA**AATCT**AC**AATCT**ATTGAGTTGCCTTATCGCTAGGTCTCCT
AGAGAGTCATAGCTTTTAAGCATTTTCAAATATTTCTCTTTTCTTTCAACAAAGCAAAGCCAGTCTCTTTCAACCTCACC
CTCACAACGGGGAGCCCCTAGTTGCCGGTTCCGGCCACGCTATCTCCTCCACTTCCCTCCTCTCTCTGATATTCCCAAAC
CATCACTCATGATTCAAACGCAAGCACAATTGATACATATTCTGTAACGCAATTATAACATGCTTAGTTCCCCGTTTAGC
GTCGCTTCCTGGCCTCCATAATTTCATGGCTTGCCAGTAATATTACTTCCCATTTCTCCCACTCAACTCCTTGACCTTTT
TTGCGTATAAAAACTCCGGCCATGCTTTGTTTGTCCACTAGGGTTTTGGTTTCTTCCTCTCTTGCTCTATTTCTTCTTCT
TGCTTTAGGGTTTTAGTTTGCTCTTGTTGATTGGAAATCATCTGGGTTTGTTGAGAGGAA**ATG**...

Prudul26A030330T1 (KNOPE2-like)

GTCCCATAATATAAGGGTAGGGTTTCAAAAAGTCGTGAAGACGTTTAACCTAAACGCACCGCATAATGAATTGTTCTACC
ACCACCGACGTCCATGTTTTCTATTAATTTTCCTTTTCAAGTTCGATTTCCATCATAGTAAAGCAACCAATTGAATTTGC
TCCCAAAATGTCAACTACACGCA**CATATATATA**TTCATGCATGCGTACACAATATAGAAAATTAATATTAATTAATGCAG
ATGCTGGTGGGTTCCAGTTCCATATGCATCTCGAGCTCATTGGGGAGGAATGCGTGCCCTCCACGTGCTAGTGTGTCCCA
CGGTGTAGTGGCCGCCTGTAGGTATGCATGGCCCACCACTAGAATTCGTCTTGACTACAT**AGATC**ATACTTTTTATCGAA
TAATTTTTCCTTTGTCGAATGCATCAATTATCAACATTATCTACCTACAACTGTACTGCAAGTTATTATTAATTCCCCAG
TAAAAATATCAAAGGTAAAACAG**TATATATA**GAAAAGTTAACAGTAAACTTTAATAGAGCCACAAATAGGGTTATACAAT
GTGAGTTGTCAATTACTATAATTGGGCAGAGTCGAAGCTTCATTTATTAGTAATGTCGTCAGCATAAGAAGCATAGGTAT
CAATGTACAATGAGTTATATACACAATTTATACAATATTATT**TATATATG**GAAAAGGAAAAGTCTCATCTTCATCTTGCC
ATTTACAAATACAAAGGCTGCTTTTTTATATTTTATGTTTTTTCTGCTGGAATTTTAAAACCTACTTTGGTCGTGTGCCT
GGAGCCTCCTCTTTCTTTTTTTTCCATGTTTGGTCAATTTAGGCATTGCATTTTTCTAATTTTATTTTGGTGTATCTAGCT
GTCAATTTGGGAGTGTTTATAAAAGGGTTAAAAGTATTTTTTGACAATAAAAAAATATTTTTAACAACTAT**AATCT**CTA**A**
ATCCTAAAATTTAAAAGTACTTCTAGGTCATAAAAGCACTTTTTGAATGTGCTTCTTTTATCCCAAATATTTTTTCTTAT
GAAAATTTATAATAAAAGCATTTGCGATCGCTACAAGTGTTCATGAACAGAAACGCCTCATAAAGAAGTAATTGAACATT
GTTTACACCTTGAAAGTAACACACACGCTAAATAA**TATATATA**TATATTTTAAACAACAACACAGTAGAAAAGGAAAAAG
ATGAAAAAGGAAGGAGCTATTATCATATCTAATCATATATCACATAGTTGTTAGTTGGGCAACAAGCACACTCCCCAAGA
ACACTGCCAGTTACCCTCTGATACTCCACTTCCAGCAAATGTTCTCTCAGTTAAAAATTTTGTGGTTAAAATGTCAAGAT
GAATAGAAATAAACAAATATACTGCCTTTTATTTTATTTTATTTTTCTCCTATAGCTGTAGCTAGAACTAGAAGAAGACA
TATAGAAAGAAAGAAAGAAGAAGAAGAAGATAAACTTTTGTTGTCTGCCATTTGAATTGG**ATG**...

Prudul26A026282T1 (KNOPE2.1-like)

GTAATGTTCAAGATGAACATGTTAATTCGAGTCAGTGGCGGAGCCAATGCATGCCTAGGGAGGGCATGTGCCCCTCCTCA
AATATTGTTGTATGGATATTTAGTTACAGTAATATTATATAAATATTAAGAAATTCCTCTTAAATTCTTAAATTTTTCTT
CTTTTTTTTTAGTTCTCTCTTTTATTCTCAAGTCTAAATTTCTTTTGAGTTGCATATATTTTATTATTATGAATGAATGT
TAGGTTATTTATACAAAAAGTAACAATAGAGAAAGTAAAAAAGTTTGAATTTTTAAATGTGCCCCATCTCACTTAAATTT
CTGGCTCTGCTACTGATTCGAGTAATTTTCTTTTGCAACTTGAACTATAACTCTTGATGAATCATTGATCATCTAACTCA
TTTCGTACACACACACCATTCATTGACATTACAAAACAAGAAAAAATTTCAATATAAAAAATGTCAAATTTCGTTCACAC
CAATTATTGGTTGTGTTGTAT**GATCC**ATTGGTATTTATGTACTGGTGCAATGCAAGGCAAGA**CATATATATATTTA**TATG
TTTAAAAAATGTAATAAAAT**GGATCT**TTTTTTTTTTTTTTTGGTCTGAATAATAAAAT**GGATC**ATGAAAATAAATAGTGT
AGATGGGTCATGCCACGCAATGGGTTGCGAATTGAGATGGCCACTTTTTGTAGTTTAGTCGAGACTCGAAAGGGAAGTGA
CTTCGACGCTCTGTGCAA**AGATT**TGGACCTAGTCATGATCAGACAACTGAAAAGAAGTTCAGCTCAGATGCACAGGGCCA
AGGCTCCAGTTTCTCCTTGTCCGTACGCCAAAATTTTACACTTCCCAATAGACCATCGCTT**CATATATA**CAGTAA**GGATC**
AATTTCTTCTTGCAAATA**AATCT**CTGTCTATAATTAGTTTCTATTTTTTTTATGAGTTAATTTTG**TATATATA**TTAGACCG
ATACAGTAATATTCAGTAATTAATTATATACTAAT**TATATTTA**CTG**TATATATG**GAGAATGAAAATAAATGCCAAAT**AGA**
TTTTCTTTTAAAAAAATTATATAGCAAAAAATTTATAACTCAAATAATTAACAACAGTTATTCATTCATCCG**AGATT**CTA
TATTAG**AATCC**CTCTTCCAACCCTATATCGTTTGCATGAAAAAAAATATATAATTTGAATTATGTTACTTTACACGATAA
AGATAAGTGACAACAAACTATTTTTTGGAAGCATTTCTCCGAGCATGGAATACTGGTTGTTGACCTTTTGACTTGATGTA
CAACTTCTACATATACATACAGCCCATTAACTACTTATAGTATAATTAATTAAACAATTAATGTGTGCGCTCTCAATGGC
CTCACACTCCCACTTTCCATGTCTATAATCACAAGCTTAGCTCTGGTCTTTTTGTCTTTCCCTAAGGCTAAGCAGATATA
TAGCTAGCTCACCCTCTTCTCCTCCTCCTCCTCCTCCTCCTCCTCTTTGGAAAGCCAACA**ATG**...

Prudul26A020729T1 (KNOPE6-like)

AGTGTCATCAACTAATTACAATTAATAAAATCACGCTCGAATAAGACGTAACGTAGATATGATGAATTTATAAAGTTATC
TTTTGATGTATGTAAGTTTTACCATTATATTCATATACATGAACAGTTAACAATTTTCTACGCCCTAAATCGATCACTCT
CGAAAATAAAATTATAGATAATTGGCTTTAGCTAACAAATGTTAGCTTGTTTGGCTAGTAAATTAAGCTTAAAG**GGATCT**
TTGAGCCCAGCTGAACAAATGCGAAAATAGTCACCCCTCCTTTTGGCTTCAATTATTAGAGT**GGATT**GCACTATCTGCTT
CTCTCAATTTACTGGGAGGAATATTCATACGGAATAGTTAAAGCTTTGGCACTTATTAATTTATCAACAACCAAGAAACA
AGAGAAAACAAAGTTAGGTTGAATACAAGGAAATGTTACGGAAGTGGAAAAAGAATAAACTAAAAAGAAAATAAACCATG
TCATCAATTTTAATATTAGGTATTCGCTCATGCGATCACATTTCTTCATCATCTGTCACATCTCTCATCCTTGTTTCTTT
TCTATCATACATTTTCCCTTCTGTCTGTCTTTGGAGCCAAAGAACAAAAAAGCAAAACTGAAAGCCAAATAAAATGCTAC
CAAACAAAACTCGCACTGGTGTAAAACTGTTCACTTCATTGATGTGAGAGAGAGGTAACATGCAGACACTAATTATTTTC
AGACTAATCATATTTTTTACCTGGGTTACTGGTTTTTTAAACTTAATTCATTCATGTG**AGATT**AAAAGTGAGAGTAGGGT
AATTCAATTT**AATCC**TCCAATTATCTGGTTCTTGTTCCCAACCACAACCAGGGCCTGTGGCGGTGAAAAGAACAGCAACA
TGAACAGGAAATGGGTGATGAGTGAGTAAGTACAAGCAAAGTCTCCTGAAACCACAGCGCTGATGTTGTTGGGCCCCAGT
GTTGCAGTCCCAGAACCAATAGGGTTTTGAAACGTAGTAGAATGTCACGTCACAGTTTAAAGGCCATTCCAAAGTCCCGA
TGCTCTGAGGAAGGTGACGGGTGGACCTCTATAAATACGGGCTACGCCTCTCTCTCCCTCTAACTTCAAATCAGACCTTT
GTTTGTTGGTCACCGTTTCAGTTCAGCCTTTTATCCGTAGTGC**AATCC**ATTTATCTCTCACACGCGACCCCAAACACAAA
CAGTTTCCTCATGTTCCTTCTCCACTTTCTCTCTGTGTCTTCTCTTTTCCTCAGGTTTCCTTGGAAAAGAAAGTAAAGTC
TGGTCCTTTTCCATGTGAGAAAGAAACCCACAAAACAAAAAAACAAATGCCACTTTGGCTCCTAGTCAAGCTAAAACCCT
TTTCTTCTCTCTCTGTCTCTCTCTGTCTCTCTCTCTCTCTCACTCACAAGCTCATTCTCCACTGTAGAAGTATTATTGTT
GCTTAATTAGCTACTTCTTTTTCTTGGACATTAGAGCTTGAATTCGAGAAAGATATATGT**ATG**...

Prudul26A030151T1 (KNOPE3-like)

TCCAAAATTTAATCACGATATTACTGGCGACAATTTT**AGATT**AAGCT**GGATT**TACTTAGAGTATTATTTCGTGTTTGAGG
TTACTATAAG**AGATT**AGGTTATTGTCCATTCCAATTGTAATAGCTATTTGAATATGTTAACCCATGTTTTTTTTTTTGTAA
GGATTA**AGATT**CTGTATCATTCTAGACAAATATCTAATTCAATTCTGATAGTTCATTTAATTTCAAATCGCATACGATTT
ATAGATGTTTAAAAAGTCTACGCAAATGGACAAAACTTTATCATCTTTATTTATTGGGGTTTCATACAAAAGTAAACATC
ATATCAGTGATTATTAAATT**AATCC**CCCATCAACGATAATAATATGCC**AAGATTTT**CATTGAGTTATTTTACTTTTTGTT
CTTTTTTCCCTATGCTAACTTCCGGTTGTGAGTTTTTTTTTAAATTAAAAATAAAATAATGCTATACTTATCATATTTTT
GTATTATATGATATGTCACATTAATTAAATCAATAAGGTATATTTTAATAAATAAATAAAAAATATTAATCACATTAATT
AACTGATGTGGTATCTACATATCAACTGCCACATCATATAATATAAAAGTGTGATAAGACTAATATTATTCCCATAAAGT
GAAAGGT**GGATT**GATAATAACTACCCCAATAGTCAGTTAAAAAAAAAAACCTACCCGAATAGTA**AATCT**CATATATTATA
TTAAATTGGAAAAAAATATATAATAAGTGGTCTTATAAAATTTCACTCATTCT**AATCC**AAACATTTTGCTTCTTAATTAA
AGA**AATCT**TTGCTACCCTTAATT**AGATT**CAAAATACCATTATTTTTCTTTGTAAAGAAATTAAAATACCATTATATTCTA
AAATCAACTATTT**GATCC**GAATTTTCTATTTTTTCAACTCCTATATCATATTGTGAAATAATAATAATAAAATATTCTTT
TACTAGTAATAGGGGACCCTTGGGCTCCTCGGCTCCTTAATTAATATTAAAACCTGCCACAA**AATCC**CACCATTTTTTTT
TCTTGCTAAAGCAGTTTCTCGAGCTCATTTTGCTCGCCTGAAAACCACTCTGTCCAAAAACCTCTCTCTCTCTAAACAGC
TCCTCAATTTCCTCATATACGTTTTTGGTACCATCC**CATATATA**AGCTCTCAAAGCTTTAACTAAACCCTTTC**AATCT**CT
CTCTCCCCAAAACTCAAAAAAGCTCGCATCTTTCTCTCTCTCTCTAAGTTTCTACACACACACCGTGTAGTTTCTCTA
CACAGAGAGATAGATATAATCA**AATCC**AGCCATATATCTTTGTCTTGTCTCTCTCTCTCTCCTATCTTCTCAGACTCTCA
GAAAGCTTCTAAATTTTCATATCCATGTCCTCAGAATCATCGTCTACAATTGCTCCTTTTTAATTTGTAAAACACAGAGA
AACAGAGTGTCCTCTCTCTCTGTCTCTGCAATTACTTTAATACCCAGCTGATCGTCGATC**ATG**...

Prudul26A023314T1 (KNOPE4-like)

TATGTATTAATCACGATTATTTCTTACAAGTCT**AGATT**GATGTGAGAATGCTAAAGATGACCAGTTCTTTTC**AATCT**TTA
TAAGTCGAGTATAGTGGTTACCGTGGAGGGTGCACGGAGTCATTGGATAATACTCAATAGGCGCTCATTTGATATGTGAA
TATCAAGTCATGAATTGAATATACTAGAATGAAAGTATATGTTACGA**AGATT**ATGTATCATGTTTTTGGTGACTTTTTCA
TATTTAGCATGATTTGTGATGAATTTCAAGTATGCATGTGTTAAGAGTCATTTAAAAGTAATTAAAAAAATTCGAATAAT
TTTTTATTTCTTAGATGTAAGTACAATATAGACAATAAAAAATTATAATCATTTAAAATATGTATGTATTAATAAATGCA
GATAAATAAAATAAAAAAAGAATGATTCCGATACCAATTATTGATAAGTAAACATATCATAACACGTAAACATACCATAG
AAGGAAGGTGAGAA**GGATT**AAATGATGCGCGCTTATCGAACCTCGTAAGGTATTGCTCTCTTGTCTATAT**AGATT**TGTTA
TGAGTGAATATTTTATTAAGCGGTGTCCTGCTATTTTGTCTTTGTCTTGTAAAGCTATGTTGTTCGAGAGCGTAACCCAA
GGGTTAAAGGAGAAAAGTTGCTTCTGACAAAATTAAAAATAAAGGAAAAAAGTTGCTGCGTCCTGCCTACTTTCCTTCCA
CTTTTCCTTCCTCTCTCTCAAGACTCAACCATTTAAAAATAATATAAACGATTTCAATGTCGACTGCTGTGCTGTCCTTC
CTGAATATGAAGGGGGTGGAAACTTGTCTCAAAATCATGTAAAGAAACTTCTTGCAACACTACAAAAACCTAACTCATAT
AA**AATCT**ATGTTATATATTATATGCCTCCAAATATTGGTACGTCAGATATCCCAAGGGGTCAAGTATGGAGCAATTAACA
ATCACAATGACAATAACGCCCCAAAATGCCACCCAACGGTCCAAAACATTTCAATAATTTTTCCATCAATTCTCACCGTT
GGATGCGTTGTGAGCTTTCTATACATCAATGGCTACTTACTATTACAAGTCTACAACACATCACAATTTTCAATCATCAA
TAAATCAATGTTTGTCTTATTTACTGGGAACGGCAACTCCCAATTACATCGCCACATGGCACTCTATCTCACCGTCAAAA
CATCCTCCTCGGTCCTGGCGTCACCCATCCATCACTCAACAAAGTCGTCGTCTCCCAAAAAAAGAGCTTCTGCACTCCTT
TAATTGCTT**AATCC**AGCATTCAAGTTTCAAACTTTTAACAGCACCACTTTCTCTCTAACTCAGAACATCACAAATTTTTT
TTTTTCCTCTCTCTCTCTCTCTCTCTCTCTCTCTCTCTCAGCTCCAAAAACTGCTTTTTTCCGAACCACGTTTTCTTG
TATGTACTGAAACTAAAAGCTCTGTGCTTTTTTTACAATTTAATTGCAAAGACCAAAGTA**ATG**...

Prudu_014112 (KNOPE7-like)

AATTCTTTTTGTCCAACAATAATAAAAATATTTGACAAAATGTGAAAATAATATTAATAATAATAATAATCAAATTGATA
TTGAAAGCATAATACACCAGCCAAAGGAGACGGCAACAGTGACAGCTCAACCCTCAGCCGACTTTCTTAAATAACAAAAC
ATATAGAAAATGTAAGGGTTTGGTACCACAAATACGGTGCCAATATTTTGATTAATGGAGCACCCTACCTATGTAGTTGG
GTGACTATTACGTTGACGTATTTGCGGTAATTCACAATGCATTATAATAGTTAAGAATTACTTACATACGTGTTGATAAA
ATATATCTTGATTCTATTTATTGTTGTATTACATCAGTCGTCAACACGCACATGAATAAGTCGTAATCGTTCATGCATGT
GTGGTAATTTTACACTATGATGATGGAAGCTTACATTTTAACATGGGTAGCATATATGGCTGGAATTGAGTATCATATAT
TGATATGCTTATTTATTATTTATCCACCTGACCTAAAAAAGTTAATAAAGTAATTGTTACACAACTTTATACAGTGTGAC
ACAAGAGCTATATCTAGAAAGCTACATATATGTAGATAGACTGTAGTGTTATCAACAATGAATTCATATTGAAAGGAGTG
CTCCATTTATGAGACTGTGAAGTGTCCAAATCAGGTCTTTAAATGGATGGTTGAAGTCTAATTTGTCATTTTAAAAGGGA
AAAAAG**AATCC**ATATTTTCTTACTGATAATTAACCAAGAATTATTGCATACCTAATCAAAATTCATTCCTGATATACCA**A**
GATTAGTTATAATTAAATACTTAACCTCCAAGAAAAACCCTTTTTCTCTGAGTAAGCTTTAAGTTGAATTAGGACCCCAG
AAAAACAAAATCAGAGGAAATTATAAAAACAAGAGGAAACAGAAAAGAAATAAGCAATCAAATTGTCAGGCACAGAAGCA
AGCAGCTTTATCTTGTTTCTTTTTATGAGCTCCCTTCCAACCGCTTTCATGGTAATGATTCATTCTATTGCCCCTGTTTC
TATTTTCATCCCTCAGTCAGTCCTCTCTAAGCTAAGAACTCATTTCTCAAGAGGCATCAAAACCATTGAAAACCTCACTA
AAAGCTTTCAAATTTTCCTTTCTCTATCTCTGTGTATGT**AAAATCTT**TGTTTTTCTCAGACGTGGTGCAGAAAGTTGTGT
GTCTC**AGATCC**CGATATACGGCTCTCCATGGCATCTTTCTTACACCATTAACGATAAAACTGTCAAAGCTATCAAGTTTG
CAACTTTCTCTCCTTCTCTCTCTCCCTTTCCCTCTCTCTCTCTCTCTCTCCACACTGGCATACACCATAATGGCATAAA**A**
ATCCATTCTCAAACACAAGGGTCTCTACTTTCTTTGTGGTCCTCAAAACCCAACTCTCACTTACCCTTTTGAAATCACAG
CTTCCTCTACAAAACCCATTTCTCAAATCACAAACTGGTTTAAATTTGGCCCTCTAGAAA**ATG**...

Prudul26A009757T1 (KNOPEM-like)

GGGCTAGTGGAGCCCAGCTCCTGCCCTAGCTAGACTTGGGCATTGGAAACC**GGATT**GAGGGCCTAGGGGGCCTCTTGCCC
GGGCTGGGGCCTCCCGGTTGAACTGCTCTTACAATCATTAGT**AGATCT**ATCTAAATGAAAACGTAGTCCA**CATATATA**AT
TTAGGGTTACTTGTTGAAATACCCTATGTGGTTTCAAAATTGTCTCAGGTTGCTCCCCTGAAGTTTATAATGACTCAACG
TCCCTCCCTGAACTTCCCTTCTGCGACCAAAGTTGCCTCATTCCGTTAGTTTTCAGAGAAAAATTGTTAAATTAGCTTAT
GTTAAAGCAATGTGGCATTTCAACTTGGAGTATATGGTCATTTCGCATATACTCACCCATCAGTTGTATTTAGCCCGCCA
ATTTGCCCCAATTCTTCCCAAAATCAAAACCCTAATTTGAGACCTGCAACAAAGTGACTAGAGAACTCTAAATTCGCGTG
TGCAATAACCAAATGATGTG**AATCC**TAAAACCAGTC**GGATCT**TGTGT**TAAATATA****GATT**ATGAACCAACTTTAACTACAA
ATT**AATCC**AT**TATATATA**TATATATCTGCCTTTGATTACCAGTTAGTTTTTTTGTATGATATACAT**TATATATA**AAGGAA
AATGGTTGAATAATAATTTATTAAAAAGCTTTTGGT**GGATC**AATTAATGTCTGGGTACGTACTTACAT**AATCT**ATGATAC
CATATATATCACAGGCACAGCAATGAAGGGTCATTTTCGTATGAGTGCATCCACAGTAGTGGAAACATTCTCTTCCTCAA
ATAAATGTGAGCAAACATTGATAAAAGAAAGCAGCCCTGATACATCAACAGTGGGGAATTTGGAAGTACTGTTTGAAAAA
GTGAG**GGATT**GACTAGTAAAAAAGGAAACTTATATAAAGACACACAATGCAGACGGATGAATAGGAGGAAATGTACTAGC
AAAGTCCCCGCTGTTCGAGAAACCCCTTTGATATGTTACTTTCTTTCGTGTATAAAGACAGAAAAAGAGAGAGAGAGAGA
GAGAGAGAGAGAGAGAGAGAGAGAGAGAGAGAGAGAGAG**AGATT**TCAAAGGCCAAAAACCCACACCCCAATTACGCATTA
GTGTTTCTGACCGACTGATCATGATGAAAGACTGATCAAATTAATATATGACATATAAATTATATGCATGCATGAGTCAT
TCCAGTGGTGAATTCCAAAAATGGGAGCGTCTCTGTACTCTCTTTGCACTGATCACTTAGTTGTAA**TATATATA**AGAGAG
AGAGAGAGAGAGAGAGAGAGAGAGAGAGAGAGAGAGTCACAGTACTATAGTAGACTAG**TATATATA**TAGTTAATGTGAAC
AAAAATGGTAAGACTGACTGGTATGATGCATAATTGTTGTTAGTGCAGTCCAGA**GGATT**TGTATTTGCTTATACTTATAG
G**TATATATA**GCTAGAGTC**GATCT**CCTCTCCAGGGTACGTTGGA**AGATT**TGTAATTATAAT**ATG**…

Prudul26A005140T1 (KNOPEM2-like)

ACTTCAGCAAGAATCGAACTCTGGCGCTACGTTAAATGGAGAAGCGCTGGAGCCAACTCCACCAGACACGCCCTTGTGCA
AATGTCTAGCACGCTATAC**TATATATA**CAATTCCTATAATATTAA**TATATATA**ATATAAAGAATTTTTTTTTCGGAGGGC
CCTTAAGAATTGGGGGCCCTGTGCGGTGGCACCACTTGCGCCACCCCAGGGCCGCCCCTGGTATACAACAACAGTACTTT
TTTTTATAATGGTTTGCAGTTGTTTGTAAACAACGATGTTGTGGTGTCACTTTAATTTTGTG**AGATT**TTA**GGATC**ATTTA
ATTCTATACGTAAAATTAGAACTAAAAATGTAAAATCGAAGCAAAAGACTCATTAGTGTAGTAGTTTAGTATATTATCAC
CTCAAGAAAATATTCATGCACTTGAGGTTTTGTTCG**AATCT**TTCTTTCCTTAATATTAGTTGAATTTAAAAAAATAAAAA
ATAAATAAAAGAATAGATAACATTGAGGATAAGATAAGATATACATTTGAGAGGGTGAAGATACTTCACCCATTAATTTC
TCATAT**TATATATATATATATATATATATA**TACACTGTGCACA**AATCC**GAAAGAACAAATAATTTGCATGTTT**CATATAT**
AACGATTTCATGTGCTACTCGGTGTTTCT**AGATT**GG**AATCC**AA**AATCT**GCATAAATTCAATCAACAAAAGGAAACGAGTG
CTCATAACTTGTTTGGTTGTTTCCGCAATGGACGCGTGCAGTTTGAATTAAGCTCCCAACTAAACCACACAAGAACATGT
ATGATGCATAAACTAGCTATTAATTTGTAACGGTTCTTTCGTCTCTGAT**GATCT**CATATATTTGTATTTGTATGTATCCC
TCTCTCTCTCTCTCTCTCTCTCTCTCTCTCTCTCTCTCTCTCTCTCTCGAGTTTCTGTTTGGTCAATGAGTGACGCAACA
AAGTAGGAAACTCCAAGTTGGTCAATGAATGACGCAAAAAAGAAAAAGAAAGAAAAATTATTGTCCAAATGTAAATGAGG
GAAAAAGAAAAAGACAAAAAAAATATAGAGCTTGCTTTGGTCTTTGGGTAAAGCAAAGTACAAAGTGAACATCAGGTATG
TTGCGAGACAGACAGCTTCGTCTTTTATCGAGGAGGAGAGAAATAAAACGTCCATGGGTTTCTTTCTCGGCATAAAAGAT
GAAGCAGAGAAACTATTTGATCAAGTCAGTAGTCGTCACTCTCCCAGCTTTTATCGTGATTTATGCATCATGCACATATC
TACATACTGATTTGCTTCACATATTTTTAAACTAGCAAAAACAACTACTGTTCATATTTGTTTTAGTACCCATAATTATT
ATTAATTATTAACACACCCACAAGGGGTGGCTACTTATCTCATCATCATCTTCTATGTGGTGTGTTTGAATTTGAGAGGG
AGAGAGAGAGAGAGAGAGAGAGAGAGAGAGAGAGAGAGAGAGAGAGAGCTGGTGAGAGAG**ATG**…
