# Supplementary material for: Prunus Knotted-like Genes: Genome-Wide Analysis, Transcriptional Response to Cytokinin in Micropropagation, and Rootstock Transformation
Source: Int J Mol Sci. 2023 Feb 3;24(3):3046. doi: 10.3390/ijms24033046 (PMC9918302; doi:10.3390/ijms24033046)
Supplement: Supplementary file 1 [file ijms-24-03046-s001.zip › Supplementary Tables/Table S5. Reference genomes of Prunus species, and datasets used in RNA-seq meta-analysis.pdf]

Table S5. Reference genomes of *Prunus* species, and datasets used in RNA-seq meta-analysis.

Species	[Source] Reference genomes	Run	BioSample	Organ/tissue	Rep
P. armeniaca	[GDR] Chuanzhihong, v1.0 (2019-11-20)	ERR5096974	SAMEA7817150	Green stems	1
	[GDR] Longwangmao, v1.0 (2021-03-11)	ERR5096975	SAMEA7817156	Vegetative buds	1
		SRR10034644	SAMN12651124	Fruits	1
		SRR5591376	SAMN07160993	Leaves	1
		SRR5591377	SAMN07160993	Leaves	2
P. avium	[GDR] Tieton, v2.0 (2020-09-29)	SRR11291063	SAMN14343298	Flower buds	1
		SRR11291064	SAMN14343298	Flower buds	2
		SRR11291065	SAMN14343298	Flower buds	3
		SRR13686248	SAMN17844929	Leaf	1
		SRR13686249	SAMN17844928	Fruits	1
		SRR13686255	SAMN17844922	Dormancy bud	1
		SRR13686257	SAMN17844933	Stems	1
P. davidiana	[GDR] Zhouxing Shan Tao, v1.0 (2020-07-09)	SRR15403591	SAMN20691626	Phloem	1
		SRR15403592	SAMN20691625	Leaf	1
		SRR15403594	SAMN20691623	Fruits	1
P. dulcis	[GDR] Texas, v2.0 (2018-10-11)	ERR7250779	SAMEA9962258	Shoot tip	1
	[GDR] Lauranne, v1.0 (2019-06-21)	ERR7250780	SAMEA9962259	Shoot tip	2
		ERR7250781	SAMEA9962260	Shoot tip	3
		SRR10189213	SAMN12855929	Fruitlets	3
		SRR10189214	SAMN12855928	Fruitlets	2
		SRR10189215	SAMN12855927	Fruitlets	1
		SRR11251356	SAMN14316608	Flower buds	3
		SRR11251357	SAMN14316607	Flower buds	2
		SRR11251358	SAMN14316606	Flower buds	1
		SRR16267428	SAMN22169166	Phloem	1
		SRR16267440	SAMN22169165	Leaf	1
P. ferganensis	[GDR] Accession 125, v1.0 (2020-08-01)	SRR15403599	SAMN20691636	Phloem	1
		SRR15403600	SAMN20691635	Leaf	1
		SRR15403602	SAMN20691633	Fruits	1
P. kansuensis	[GDR] Honggengansutao 1#, v1.0 (2020-08-03)	SRR15403604	SAMN20691631	Phloem	1
		SRR15403605	SAMN20691630	Leaf	1
		SRR15403607	SAMN20691628	Fruits	1
P. mira	[GDR] 2010-138, v1.0 (2020-07-14)	SRR13515756	SAMN17517089	Leaf	1
	[GDR] 2010-138, v2.0 (2021-09-23)	SRR13515757	SAMN17517088	Leaf	2
		SRR13515759	SAMN17517087	Phloem	1
		SRR13515760	SAMN17517086	Phloem	2
		SRR13515763	SAMN17517083	Fruits	1
		SRR13515764	SAMN17517082	Fruits	2
P. mume	[NCBI] BJFU1210120008, v1.0 (2014-02-28)	SRR13961800	SAMN18309663	Flower buds	1
		SRR13961801	SAMN18309664	Flower buds	2
		SRR13961802	SAMN18309665	Flower buds	3
		SRR542478	SAMN01120304	Buds	1
		SRR542479	SAMN01120305	Leaf	1
		SRR542481	SAMN01120307	Stems	1
		SRR542482	SAMN01120308	Fruits	1
P. persica	[GDR] Lovell, v2.0.a1 (2015-01-15)	SRR10432653	SAMN13266932	Vegetative buds	1
		SRR10432654	SAMN13266931	Vegetative buds	2
		SRR10432661	SAMN13266930	Vegetative buds	3
		SRR13327397	SAMN17141584	Shoot	1
		SRR13327417	SAMN17141583	Shoot	2
		SRR13327418	SAMN17141582	Shoot	3
		SRR13515747	SAMN17517077	Leaf	2
		SRR13515748	SAMN17517076	Leaf	1
		SRR13515753	SAMN17517071	Fruits	2
		SRR13515758	SAMN17517070	Fruits	1
P. salicina	[GDR] Sanyueli, v2.0 (2020-10-10)	SRR10235677	SAMN12920318	Branch	1
		SRR10235679	SAMN12920316	Leaf	1
		SRR12234088	SAMN15500014	Flower buds	1
		SRR12234102	SAMN15500016	Flower buds	2
		SRR12234103	SAMN15500015	Flower buds	3
		SRR14455846	SAMN18917931	Fruits	1
		SRR14455847	SAMN18917931	Fruits	2
		SRR14455848	SAMN18917931	Fruits	3
