# Supplementary material for: Prunus Knotted-like Genes: Genome-Wide Analysis, Transcriptional Response to Cytokinin in Micropropagation, and Rootstock Transformation
Source: Int J Mol Sci. 2023 Feb 3;24(3):3046. doi: 10.3390/ijms24033046 (PMC9918302; doi:10.3390/ijms24033046)
Supplement: Supplementary file 1 [file ijms-24-03046-s001.zip › Supplementary Tables/Table S6. Media combinations for Gisela 6 leaf explant regenerations..pdf]

Table S6. Media combinations for Gisela 6 leaf explant regenerations.

Parameters	Media combinations[1]			
	RM1/RM1	RM2/RM2	RM1/RM1+HYG	RM1/RM1+PPT
Explants forming callus	46/50 (92%)	45/50 (90%)	2/50 (4%)	1/50 (2%)
Regeneration Frequency	6/50 (12%)	2/50 (4%)	2/50 (4%)	1/50 (2%)
Shoots per regenerating explant	4.3±0.5	4.5±0.7	2.0±1.4	1.0±0.0

1, RM1 and -2, regeneration media (further details are in material and methods). HYG10 mg/L; PPT, 25 mg/L
