# Supplementary material for: Prunus Knotted-like Genes: Genome-Wide Analysis, Transcriptional Response to Cytokinin in Micropropagation, and Rootstock Transformation
Source: Int J Mol Sci. 2023 Feb 3;24(3):3046. doi: 10.3390/ijms24033046 (PMC9918302; doi:10.3390/ijms24033046)
Supplement: Supplementary file 1 [file ijms-24-03046-s001.zip › Supplementary Tables/Table S7. Regeneration and transformation frequencies of Gisela6 clones.pdf]

Table S7. Regeneration and transformation frequencies of Gisela 6 clones.

**Cassettes**	**Vector**	**Res.** [1]	**Regeneration frequency**[2]		**T-DNA insertion**[3]		**FPC**[4]		**Rev**[5]
			1st round	2nd round	TF	bands	TP	TE	
35S:m5gfp	*pCAMBIA1302*	HYG	9/231(3.9%)	6/231 (2.6%)	6/231 (2.6%)	multiple	6	6	0
35S:KNOPE1	*pBA002*	PPT	7/519 (1.3%)	3/519 (0.6%)	3/519 (0.6%)	multiple	6	2	1

1, Antibiotic resistance: HYG, hygromycin; PTT, phosphinothricin.

2, Number of regenerating explants out of agro-infected ones (and percentage) for first and second rounds of antibiotic selection.

3, Insertion data are based on Southern blot analyses (see Figure 9S): TF, transformation frequency; bands, number of bands from blot.

4, FPC, fully propagated clones from the primary transgenic clones: TP, transgene presence by genomic DNA PCR; TE, transgene expression by RT-PCR for *KNOPE1* and by microscope for GFP plants

5, REV, revertant phenotype
