# Supplementary material for: Prunus Knotted-like Genes: Genome-Wide Analysis, Transcriptional Response to Cytokinin in Micropropagation, and Rootstock Transformation
Source: Int J Mol Sci. 2023 Feb 3;24(3):3046. doi: 10.3390/ijms24033046 (PMC9918302; doi:10.3390/ijms24033046)
Supplement: Supplementary file 1 [file ijms-24-03046-s001.zip › Supplementary Tables/Table S8. Primers for qRT-PCR in CK assays.pdf]

Table S8. Genes and primers for qRT-PCR in CK assays

			Arabidopsis thaliana best hits (BlastP)[1]				
**Gene**	**Name**	**Primer pairs (5'>3')**	**Subject**	**Description**	**ID%**	**Pos%**	**E-value**
STMlike1	*Prupe.3G212100*	Fw: GAAGTGGGTCTCCAGAAGAGGTTG<br>Bw: GCTTCTGAGCTTCCGATGGATAGG	AT1G62360.1	STM | SHOOT MERISTEMLESS	66	78	2.00E-107
STMlike2	*Prupe.4G015200*	Fw: GCAAGTACAGTGGATATCTGGGCAGT<br>Bw: CAGAGCAATGTGCGAGAGATATC	AT1G62360.1	STM | SHOOT MERISTEMLESS	83	89	9.00E-140
KNOPE1	*Prupe.1G416700*	Fw: GCTTGGCAACAATAATGCTCCTCC<br>Bw: TCCAAGCCTGTCGACTCCGC	AT4G08150.1	KNAT1, BP | KNOTTED-like from Arabidopsis thaliana, BREVIPEDI-CELLUS	66	79	1.00E-118
KNOPE2	*Prupe.1G249600*	Fw: CCTTCCGTGGGGAGCATC<br>Bw: GGGTTCTAATGGAGGAGGCG	AT1G23380.1	KNAT6 | KNOTTED1-like homeobox gene 6	62	76	2.00E-130
KNOPE2.1	*Prupe.5G171100*	Fw: CGGATGCTGCTTCGGCTGTG<br>Bw: CGTGCTCTTCGCCTGTTCGTG	AT1G23380.1	KNAT6 | KNOTTED1-like homeobox gene 6	60	68	4.00E-90
KNOPE6	*Prupe.6G312900*	Fw: ATGCTCTTGTTTCAAACGGAGGG<br>Bw: AGTCCCAGCTGGTTTTTGGG	AT1G23380.1	KNAT6 | KNOTTED1-like homeobox gene 6	56	70	3.00E-76
KNOPE3	*Prupe.1G486200*	Fw: GGATGAATGGAGAAAAGGAAACG<br>Bw: GGAACCATGCTCAGGTCATTG	AT5G25220.1	KNAT3 | KNOTTED1-like homeobox gene	72	79	0.00E+00
KNOPE4	*Prupe.7G254700*	Fw: CTCTCTCTCTTTCTCTCAGC<br>Bw: GCCATTACTTTGGTCTTTGC	AT5G11060.1	KNAT4 | KNOTTED1-like homeobox gene 4	81	88	1.00E-149
KNOPE7	*Prupe.5G088100*	Fw: GGCAAACTTCCTGGTGACACTACTAC<br>Bw: ACCGCCAAGGTCACAAAACAGC	AT1G62990.1	KNAT7 | KNOTTED-like homeobox of Arabidopsis thaliana 7	85	91	9.00E-171
KNOPEM	*Prupe.5G236700*	Fw: GCTCACTTGGATTGTTTGAAGGTCG<br>Bw: CGTTTGCTGCGGTTCCTCCA	AT1G14760.2	KNATM | KNOX Arabidopsis thaliana meinox	45	60	7.00E-21
PpBEL1	*Prupe.5G104600*	Fw: CAATGGTGGAGGCGTGTCG<br>Bw: ATTGAGCAGTGTTTCGCCTATCC	AT5G41410.1	BEL1, BELL 1	52	65	6.00E-142
PpBLH1	*Prupe.7G094300*	Fw: GCAGCAGGCAAGATTGAAGG<br>Bw: GAAAAGCCAAGCACGAAGTACG	AT2G35940.3	BLH1 | BEL1-like homeodomain 1	51	62	9.00E-168
PpBLH2	*Prupe.7G146900*	Fw: GCACAAACTCCAACGCCTACA<br>Bw: TCCTCGGAAGCCAGCCTATC	AT4G36870.2	SAW1, BLH2 | BEL1-like homeodomain 2	82	90	4.00E-142
PpBLH3	*Prupe.1G373600*	Fw: ACAAGGTGGCTGGGAACGG<br>Bw: TGAAATGCCCTCTGCTGTCTGA	AT1G75410.2	BLH3 | BEL1-like homeodomain 3	49	63	9.00E-96
PpBLH5	*Prupe.6G144900*	Fw: TCCGCCTCCAACAACCCA<br>Bw: TCACGCTCGGCAGGAAATG	AT2G35940.3	BLH1 | BEL1-like homeodomain 1	43	56	3.00E-142
PpBLH6	*Prupe.8G089800*	Fw: TCTGAGAACGCACCTCAAACG<br>Bw: CCTAAACACGCAGTCCAAAGC	AT4G34610.4	BLH6 | BEL1-like homeodomain 6	65	73	8.00E-120
PpBLH8	*Prupe.6G105100*	Fw: TGGTTCATAAACGCCAGAGTGC<br>Bw: CCCCATCAGTCCACCGTCC	AT2G27990.1	BLH8, PNF | BEL1-like homeodomain 8	42	54	3.00E-89
PpCAT1	*Prupe.5G011300*	Fw: CCATGAGATTCGCAGCGTTTG<br>Bw: CCATAATCCCAAATGACAATCCG	AT1G20630.1	CAT1 | catalase 1	88	95	0.00E+00
PpCAT2	*Prupe.5G011400*	Fw: TCATCAGTAAGGCAGGGAAAGTACAC<br>Bw: TGGGCAGGGTTAAACGCAAG	AT4G35090.2	CAT2 | catalase 2	84	94	0.00E+00
PpCKX6	*Prupe.2G026700*	Fw: TATTGGTGGTACTCTGTCTAATGCTGG<br>Bw: ATGATGCCAAACTGACCAAGCC	AT3G63440.1	CKX6 | cytokinin oxidase/dehydrogenase 6	69	83	0.00E+00
PpARR12	*Prupe.1G391400*	Fw: GATTACCTGCTAAAACCCGTCCG<br>Bw: ATTTCCCGTGCCCGCTGA	AT2G25180.1	ARR12 | response regulator 12	61	73	4.00E-130
PpHK1	*Prupe.7G170700*	Fw: CCTGTCACGGGTGAGAAGATAGGA<br>Bw: GTAACACCCACAACAGCCACTATGC	AT2G17820.1	HK1 | histidine kinase 1	66	79	0.00E+00
PpRPII	*Prupe.8G132000*	Fw: GGTCAAAATCCGTGAAATGCG<br>Bw: GGATGAGTCCGAGCCTGTGGG	AT2G15430.1	NRPB3 | Non-catalytic subunit of nuclear DNA-dependent RNA poly-merases II, IV and V	81	92	0.00E+00

1, BlastP analysis against TAIR protein db. The percentages of identical residues (ID%), and of conservative substitutions (Pos%) were also reported.
