# Supplementary material for: Prunus Knotted-like Genes: Genome-Wide Analysis, Transcriptional Response to Cytokinin in Micropropagation, and Rootstock Transformation
Source: Int J Mol Sci. 2023 Feb 3;24(3):3046. doi: 10.3390/ijms24033046 (PMC9918302; doi:10.3390/ijms24033046)
Supplement: Supplementary file 1 [file ijms-24-03046-s001.zip › Supplementary Tables/Table S9. Primers for Gisela6 transformation.pdf]

Table S9. Primers for Gisela 6 transformation

Task	Target	ID	Primer pair (5′-3′)[1]	Size (bp)
Cloning	*KNOPE1*	DQ358050	Fw: GTCTAGAGTTTGGCTTCACTTCCTCGC<br>Bw: GCTCGAGGCAGTTTGCTTTGTCTTTTGG	1508
Transgene detection	*35S:KNOPE1:tNOS*	-	Fw: CGACAGTGGTCCCAAAGATG<br>Bw: GCGCGATAATTTATCCTAG	2001
Expression	*KNOPE1*	DQ358050	Fw: GTTTGGCTTCACTTCCTCGC<br>Bw: GCTTGCCCTCCCATAGTTGCC	326
	PpRPII	ppa008812m [a]	Fw: GGTCAAAATCCGTGAAATGCG<br>Bw: GGATGAGTCCGAGCCTGTGGG	191

1, Restriction sites used for *KNOPE1* cloning into the pBA002 vector are underlined: thin line, XbaI; bold line, XhoI.
